# Mechanistic insights into the acidic wet-chemical silicon etching in HF–HX–X_2_ solutions, X = Cl, Br, I

**DOI:** 10.1039/d6ra05037a

**Published:** 2026-08-21

**Authors:** Nils Schubert, Niklas Zomack, André Stapf, Edwin Kroke

**Affiliations:** a Institute of Inorganic Chemistry, Technische Universität Bergakademie Freiberg Leipziger Str. 29 D-09599 Freiberg Germany; b Centre for Efficient High-Temperature Material Conversion, Technische Universität Bergakademie Freiberg Winklerstr. 5 D-09599 Freiberg Germany edwin.kroke@chemie.tu-freiberg.de

## Abstract

Alkaline, wet-chemical silicon etching is an essential part in the manufacturing of silicon solar cells, because the anisotropy of the etching process enables texturization of the wafer surface. Anisotropic etching in alkaline systems is traditionally attributed to the nucleophilic attack of OH^−^ on Si–H, forming a pentacoordinated Si-atom as a transition state. This leads to (111) crystal planes being etched much slower, thus generating pyramidal structures on (100) wafer surfaces. In aqueous HF solutions with halogens as oxidants, similar surface structures are observed, even though the reactants are completely different from the alkaline solutions. The data show that in HF–HCl–Cl_2_ solutions, the presence of Cl_3_^−^ plays a decisive role. Trihalide ions and hydroxide ions are very similar: both are negatively charged, nucleophilic and relatively small. This suggests a similar reaction behavior. In this article, it is shown that trihalides in acidic wet-chemical silicon etching systems represent a functional analogy to hydroxide ions in alkaline systems and are responsible for anisotropic etching. The findings are supported by spectroscopic and surface analytical data.

## Abstract 2

Anisotropic etching in halogen-containing HF solutions is caused by tri- and polyhalide ions, thus representing a functional analogy to hydroxide ions in alkaline systems. Conversely, HF solutions containing only the halogen polish silicon surfaces.

## Graphical abstract

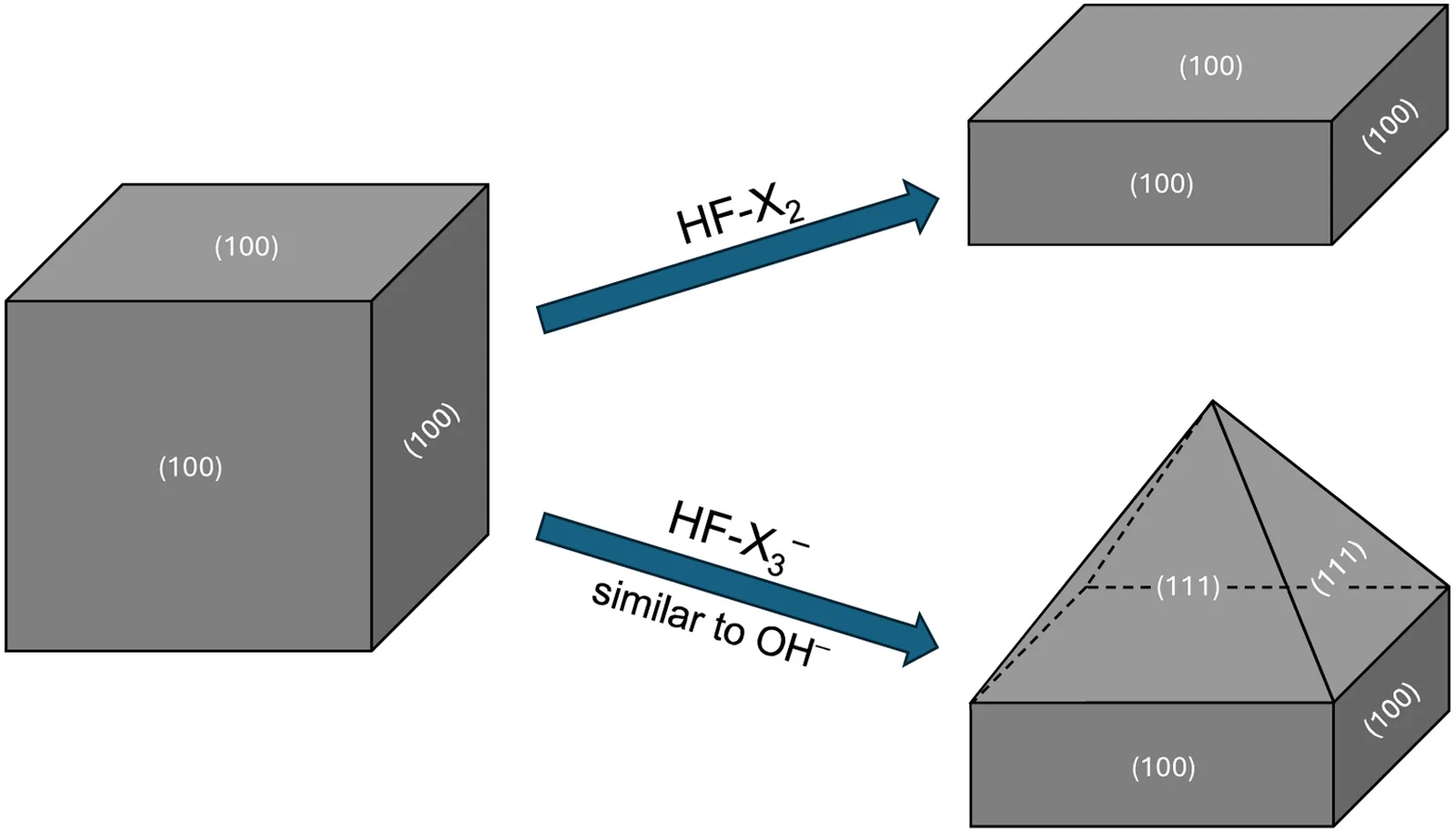

## 1. Introduction

### 1.1. Anisotropic alkaline etching of monocrystalline Si

For the industrial application of silicon substrates, Si surfaces have to be etched to achieve a desired morphology, *e.g.* for photovoltaics or microelectronics.^1^ Wet-chemical processes are used in mass-production of *e.g.* solar cells or microchips, because of their low cost, ease of use and high throughput. Silicon can be dissolved isotropically or anisotropically, depending on the chemicals used and the process parameters.^2,3^ Isotropic silicon etching is generally observed with HF solutions, resulting in polished, mirror-like surfaces.^1^ For anisotropic etching, alkaline etching agents and additives are used, leading to surface texturization with pyramidal structures.^2^ However, we recently demonstrated that it is also possible to apply silicon wafers textured using acidic HF–HCl–Cl_2_ etching mixtures.^4^ In alkaline and HF-containing solutions, silicon surfaces are mainly H-terminated.^1^ A termination with hydrogen passivates the surface, because the Si–H-bond is only very weakly polarized.^5^ The generation of pyramidal structures is derived from the cubic diamond crystal structure of silicon, as anisotropic etchants like strong bases dissolve (100) and (110) planes faster than (111) planes.^1^ Depending on the ratio of the dissolution speeds in the (100) and (110) direction, upright (tip points away from the surface) or inverted pyramids (tip points into the surface) are formed.^6^Fig. 1 shows SEM images of upright and inverted pyramidal textures.

**Fig. 1:**
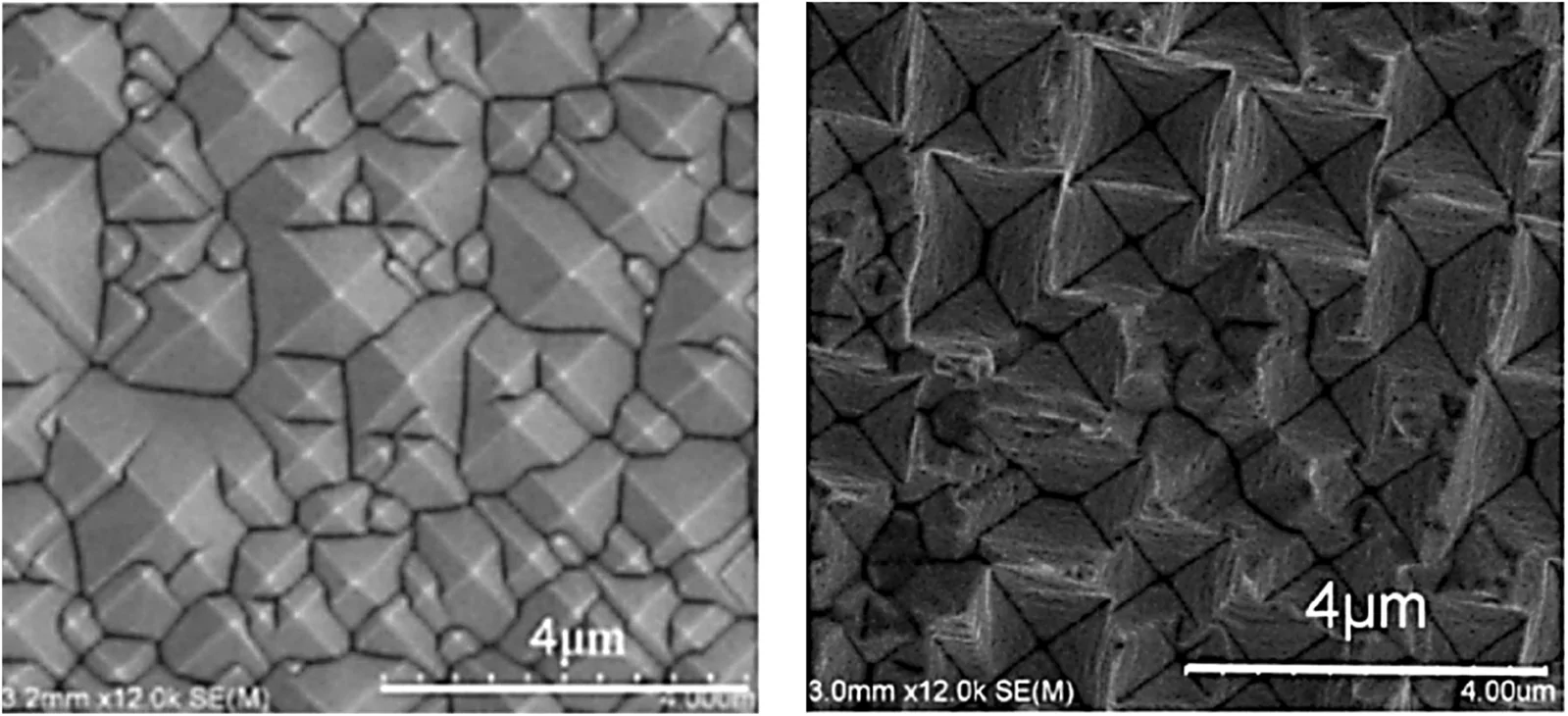
Upright (left) and inverted pyramidal textures (right) on (100) Si wafer surfaces. Reproduced with permission from ref. 2 and 3. Copyright 2025 & 2022, Elsevier.

During alkaline wet-chemical silicon etching, both water molecules and hydroxide ions are involved in the dissolution process.^7^Fig. 2 shows a chemical surface reaction model of the alkaline silicon dissolution. As seen in Fig. 2, the silicon surface atoms are attacked nucleophilically by hydroxide ions. Hydridic hydrogen on the Si–H-surface is cleaved off, immediately reacting to molecular H_2_ with protons from water molecules. Thus, the oxidizing agent in the alkaline etching are protons from the solution. The rate-determining step is the first nucleophilic attack and the heterolytic cleavage of the Si–H bonds.^8^ This is attributed to the fact that the highly inert Si–H bond is only very weakly polarized. As a result, the silicon surface atoms are only very weakly positively polarized, which reduces the driving force for a nucleophilic attack. The anisotropic etching process is reaction-controlled.^1^ A sufficiently high mixing and circulation speed of the etching solution eliminates diffusion control, which is a requirement for the anisotropic silicon dissolution.

**Fig. 2:**
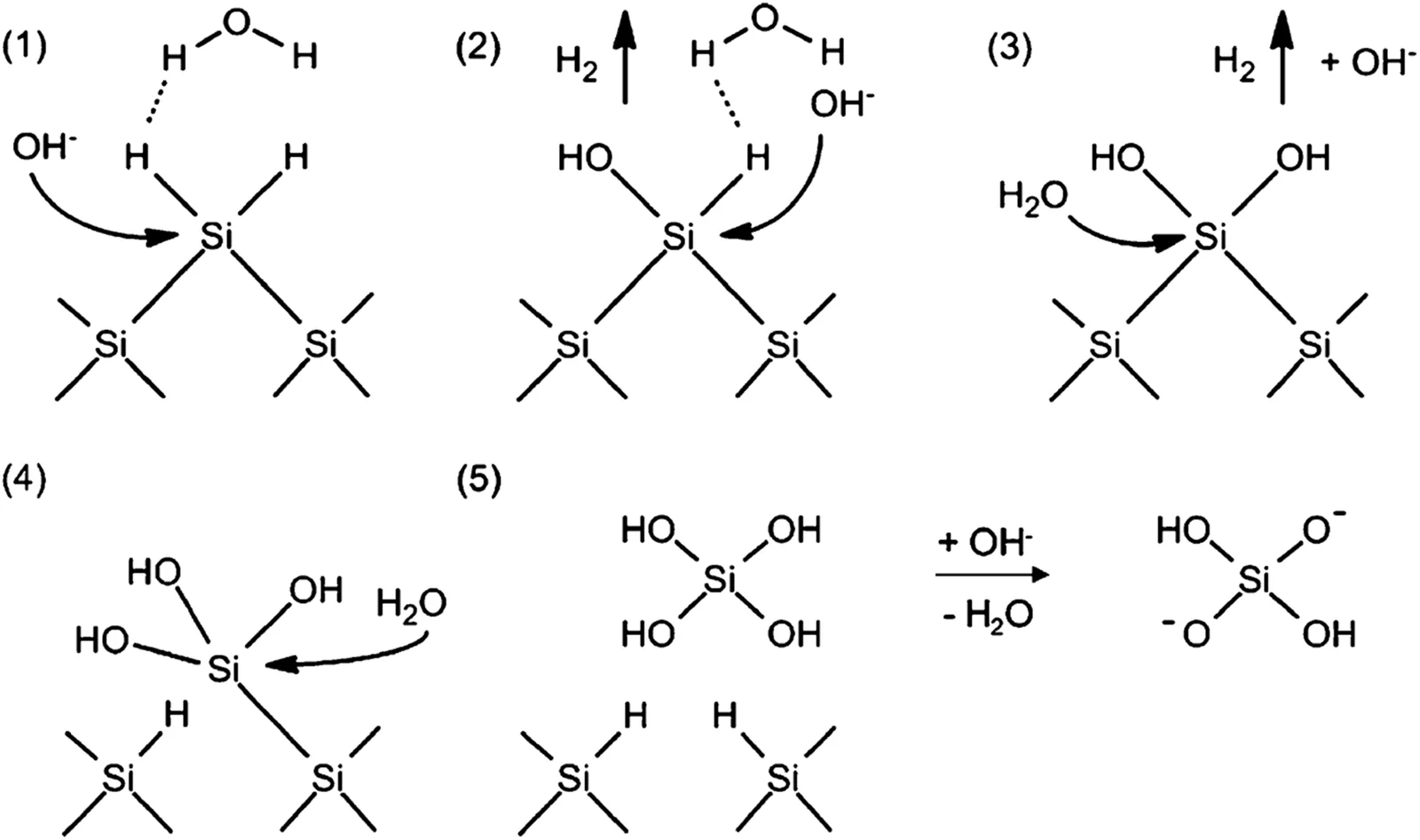
Reaction scheme proposed for the chemical dissolution of (100) Si surfaces in alkaline solutions. Taken from ref. 1.

### 1.2. Proposed reasons for etching anisotropy

The exact reaction mechanism and the reasons for anisotropic wet-chemical silicon dissolution are still discussed and several explanation attempts were made in the past.^1,9–11^

#### 1.2.1. Steric effects

Buchholz attributed the variation in dissolution speeds to varying atomic distances between the terminal silicon atoms on the respective crystal plane (see Fig. 3).^9^ With an ionic radius of 1.23 Å, hydroxide ions are sterically hindered in attacking silicon atoms on (111) planes.^12^ This results in higher activation energies for the initial attack in (111) direction, leading to slower dissolution rates compared to (100) and (110) surfaces.^9^

**Fig. 3:**
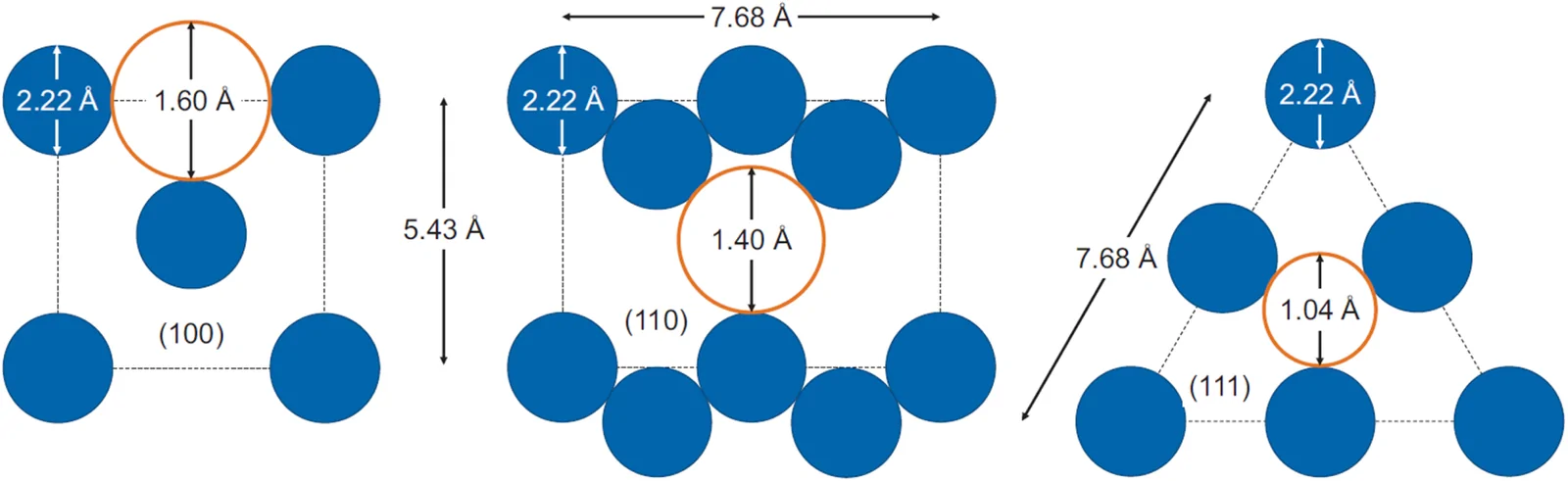
Radii of and distances between silicon surface atoms on (100), (110), and (111) surfaces. Taken from ref. 1.

#### 1.2.2. Conformational change

The initial oxidative attack by hydroxide ions results in the formation of a pentacoordinated Si-atom as a transition state, which ideally takes on a trigonal-bipyramidal conformation.^1^ Therefore, the existing bond angles have to change to accommodate a fifth bonding partner. Dangling bonds are free to change their relative position, while rearward Si–Si bonds are held in place because of the crystal lattice. On (100) planes, terminal silicon atoms are connected to the crystal lattice *via* two Si–Si bonds, leaving two dangling bonds which are saturated with heteroatoms (*e.g.* hydrogen).^13^ Terminal silicon atoms on (111) planes are characterized by three Si–Si bonds and one dangling bond.^14^ This results in a higher activation energy for the formation of a pentacoordinated silicon atom in (111) direction, since the transition state has a higher energy level.^1^ The formation of a trigonal bipyramid is very difficult on (111) crystal planes, because the 180° angle between the axial positions cannot be formed (see Fig. 4).

**Fig. 4:**
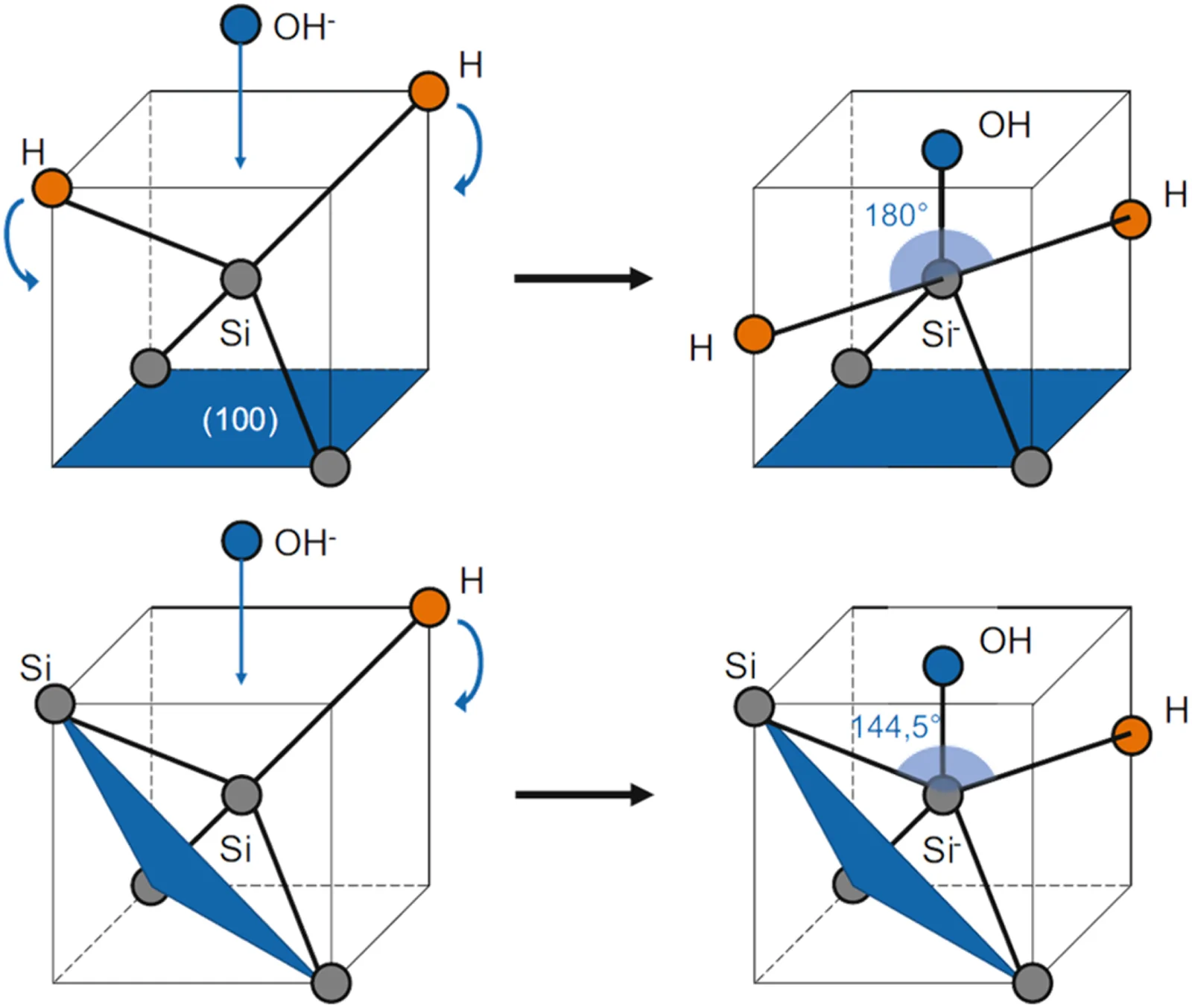
Comparison of the formation of pentavalent transition states on silicon (100) and (111) surfaces during anisotropic treatment. Taken from ref. 1.

#### 1.2.3. Polarization

During alkaline etching, the terminal hydrogen atoms are replaced by more electronegative OH-groups. This substitution induces a polarization effect on the rearward Si–Si bonds. Activation of the before only weakly polarized or unpolarized Si–Si bonds enables an attack by H_2_O (alkaline etching, see Fig. 2, step 3 & 4) or HF (acidic etching), effectively dissolving the silicon atom.^1^ Because more dangling bonds are available on (100) planes (see Fig. 4, top left), the rearward Si–Si bonds are strongly polarized on (100) planes, followed by (110) and (111) planes. In (111) direction, one dangling bond is polarizing three back bonds (see Fig. 4, bottom left), showing the weakest polarization effect. As a result, the reactivity of polarized Si–Si bonds is highest on (100) planes due to their strong polarization, followed by (110) and (111) planes.

#### 1.2.4. Orientation

In the (100) and (111) directions, all Si–Si bonds protrude into the crystal lattice, while in the (110) direction, two of the three Si–Si bonds are in plane and can therefore be attacked more easily by reaction partners.^15^ Attacking the rearward Si–Si bonds is therefore much easier on (100) and (110) planes compared to (111) planes.

#### 1.2.5. Surface energy

Etching becomes easier as the number of available electrons on the silicon surface increases.^15,16^ The electronic properties of the various crystal planes differ due to variations in surface bond density. On (100) planes, the density of surface bonds is highest at 1.36 × 10^15^ cm^−2^, followed by (110) planes at 0.96 × 10^15^ cm^−2^ and (111) planes at 0.78 × 10^15^ cm^−2^.^15^ If the near-surface Si–Si bonds of the (110) surface are included, the density of surface bonds is 1.96 × 10^15^ cm^−2^, exceeding that of the (100) surface.^15^

### 1.3. Anisotropy in acidic etching of Si surfaces

In recent years, our working group focused on silicon etching with HF solutions and halogens as oxidants. Surprisingly, anisotropic etching was observed with a lot of different etching solutions.^17–20^ First evidence was observed when (100) Si wafers were etched in HF–HCl–(NH_4_)_2_S_2_O_8_ and HF–HCl–O_3_ mixtures.^17,21^ SEM measurements indicate the formation of upright pyramidal structures (see Fig. 5), formerly only known from alkaline etchants. Because the silicon oxidation by ammonium peroxodisulfate and ozone is kinetically inhibited, the fast dissolution rates were attributed to the formation of chlorine in the etching solution.^17,21^ This led to a detailed investigation of (100) silicon wafer etching in HF–HCl–Cl_2_ solutions, where random inverted pyramidal structuring was observed (see Fig. 6).

**Fig. 5:**
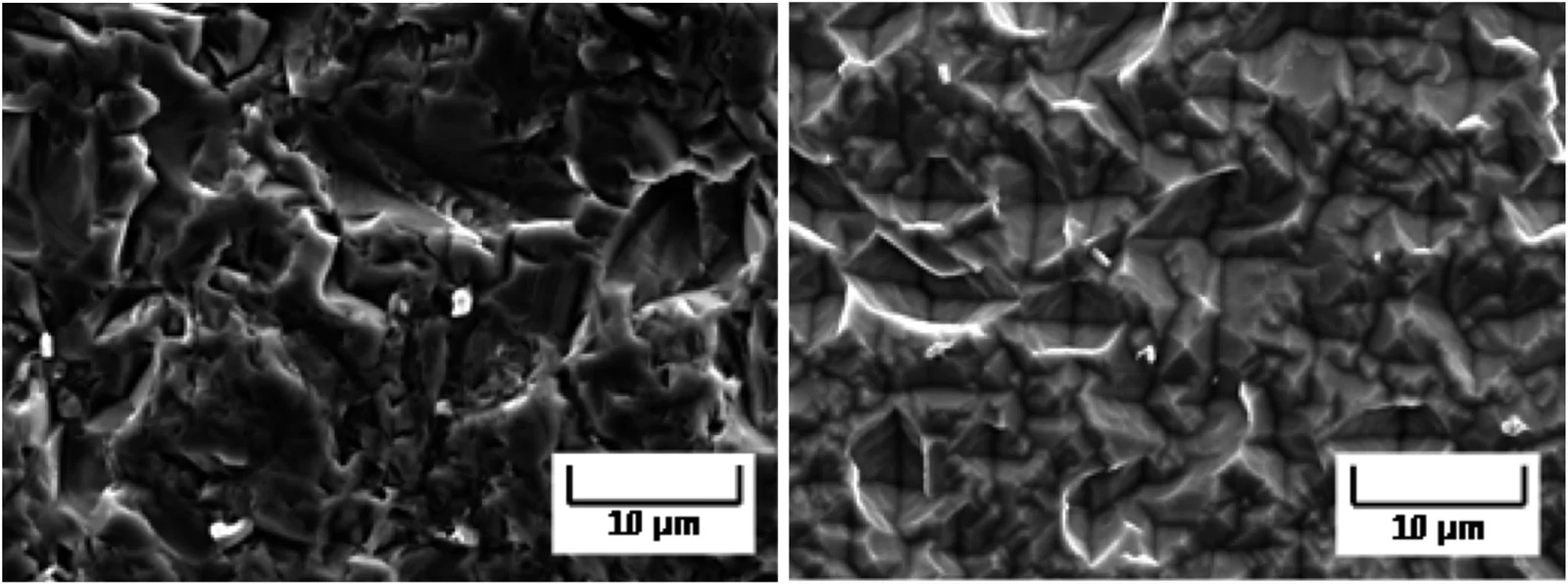
SEM images of a SiC-slurry sawn as-cut (100) Si surfaces before (left) and after (right) treatment in a HF–HCl–O_3_ mixture (*c*(HF) = 2.0 mol L^−1^, *c*(HCl) = 9.2 mol L^−1^, *c*(Cl_2_(aq)) ≈ 7.0 mmol L^−1^); silicon removal: 3.8 µm; 20 °C. Taken from ref. 21.

**Fig. 6:**
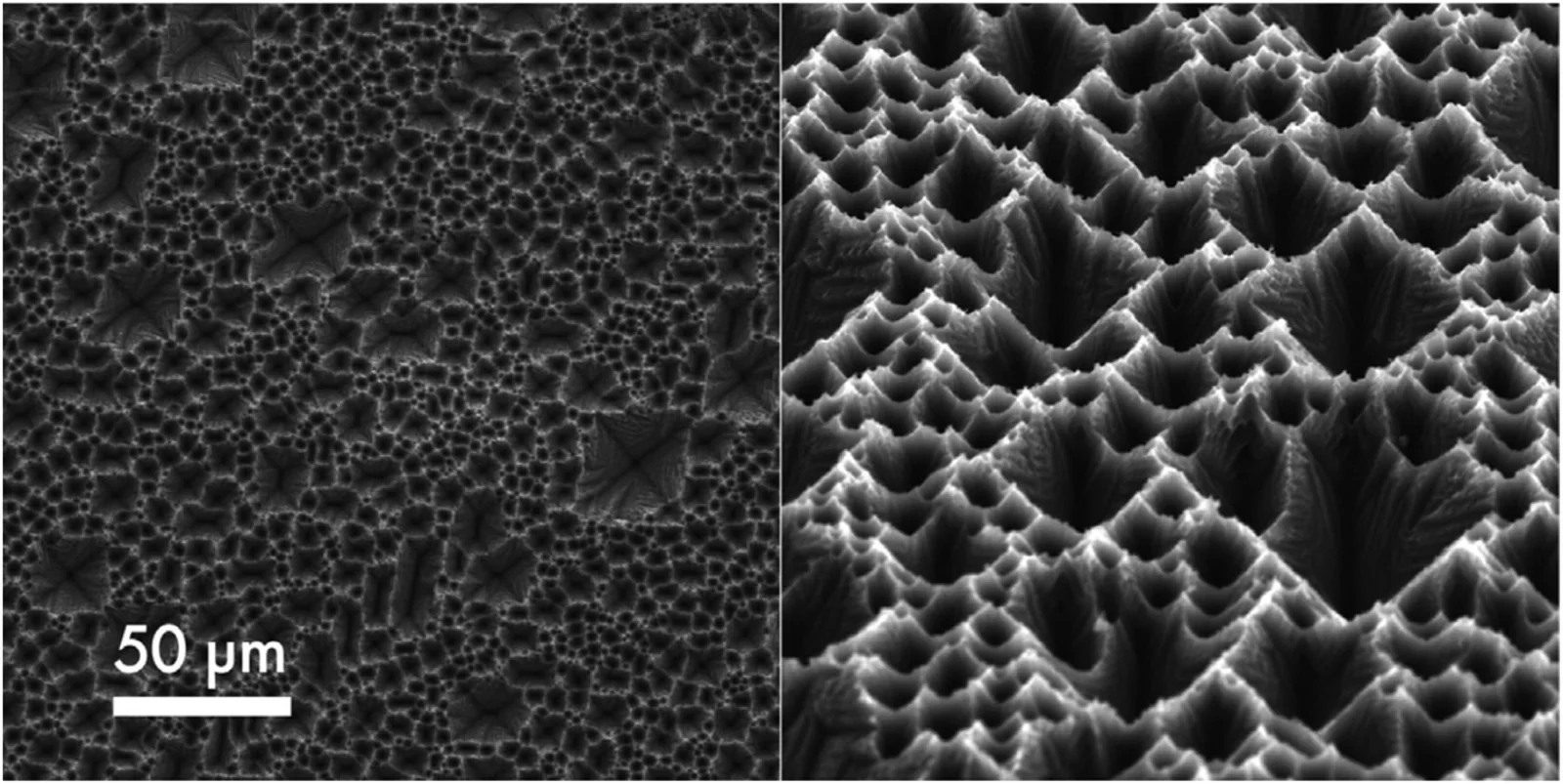
SEM images of a diamond wire-sawn silicon wafer surface textured in a stirred HF–HCl–Cl_2_ mixture, HF [50%]: 20 vol%, HCl [37%]: 80 vol%, Cl_2_: 40 mL min^−1^, 20 min, room temperature. Taken from ref. 22.

Texturing of (100) Si wafer surfaces is observed with a lot of other halogen-containing HF solutions, *e.g.* HF–HCl–HClO_3_, HF–HBr–Br_2_, HF–HBrO_3_–Br_2_, HF–H_2_SO_4_–ClO_2_, HF–I_2_–C_2_H_5_OH, HF–NaI–I_2_–C_2_H_5_OH, HF–HI–I_2_–C_2_H_5_OH, HF–HIO_3_–C_2_H_5_OH, HF–HI–HIO_3_–C_2_H_5_OH.^6,18–20,23,24^ Altered halogen-containing etching solutions can also be anisotropic etchants for silicon, although the etching process is isotropic when freshly prepared mixtures are used, *e.g.* HF–Br_2_, HF–HBrO_3_, HF–HBrO_4_, HF–I_2_.^18,19,23,25^

### 1.4. Proposed mechanisms for the surface reactions in acidic solutions

Stapf *et al.* proposed a reaction scheme for the anisotropic silicon dissolution in HF–HCl–Cl_2_ solutions (see Fig. 7).^26^ The reaction scheme involves the nucleophilic attack of chloride ions on terminal silicon atoms, forming a pentavalent transition state comparable to models for alkaline etching.^26^ Si–H is abstracted by molecular chlorine, thus the silicon atoms are temporarily Cl-terminated before chlorine is either substituted by fluorine or the Si–Si bonds are broken because of the polarization effect.

**Fig. 7:**
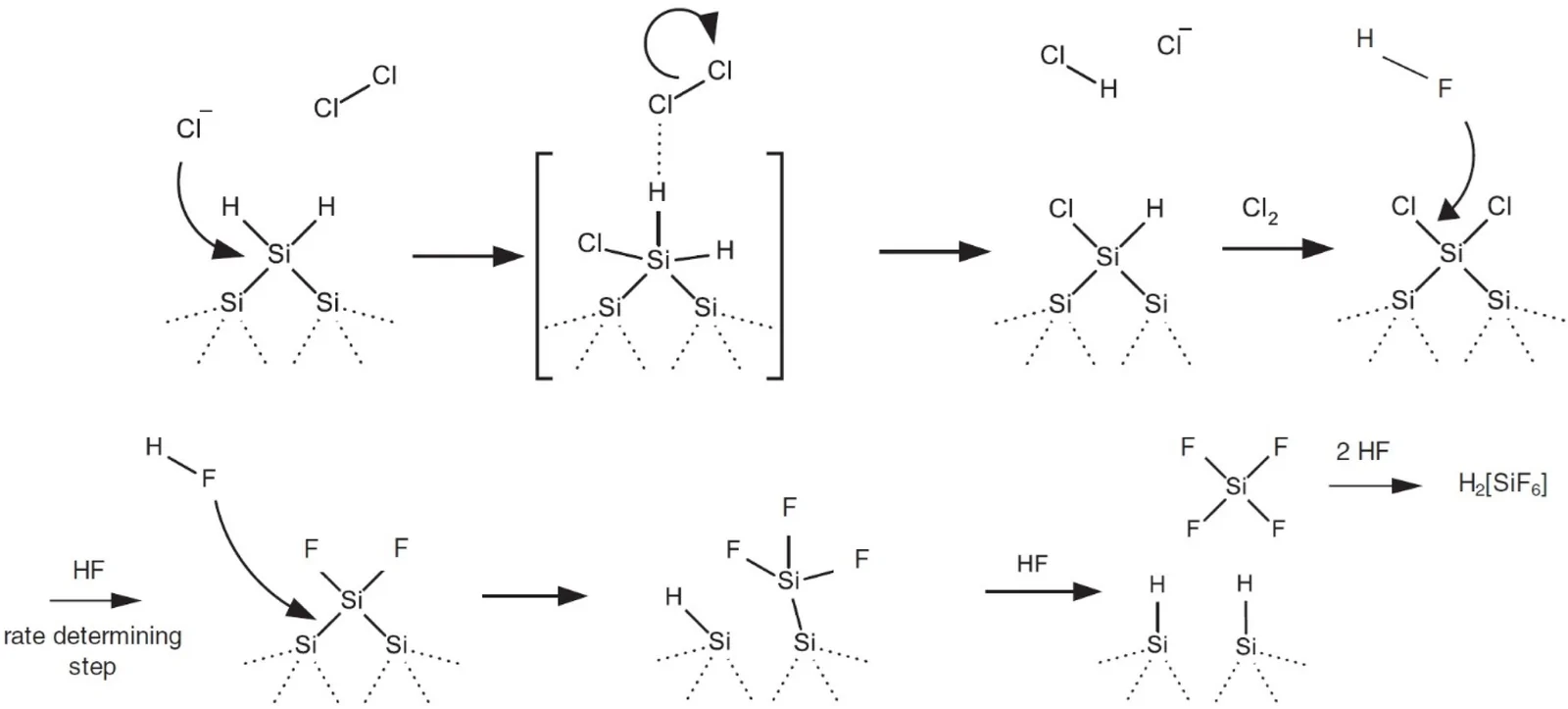
Reaction scheme for the anisotropic dissolution of silicon in chlorine-containing hydrofluoric acid solutions, proposed by Stapf *et al.* Taken from ref. 26.

Consequently, for an anisotropic dissolution process in HF solutions, the halogen and the corresponding halide must be present in the etching solution.^26^ The fact that an anisotropic etching process only occurs in HF–HCl–Cl_2_ solutions with very high HCl contents contradicts this assumption.^4^ Wang *et al.* attributed the anisotropic etching behavior in HF–HI–I_2_ solutions to the formation of tri- and polyiodide ions.^23^ Using freshly prepared HF–I_2_–C_2_H_5_OH solutions, they studied the extent of pyramid formation, which correlates with the etching time (see Fig. 8).^23^ Short etching times result in polished (100) surfaces, while small onsets of texturization become apparent. With longer etching times, more and more pyramids are generated.^23^ This assumption is supported by our investigations in HF–Br_2_ solutions, showing similar etching behavior.^18^

**Fig. 8:**
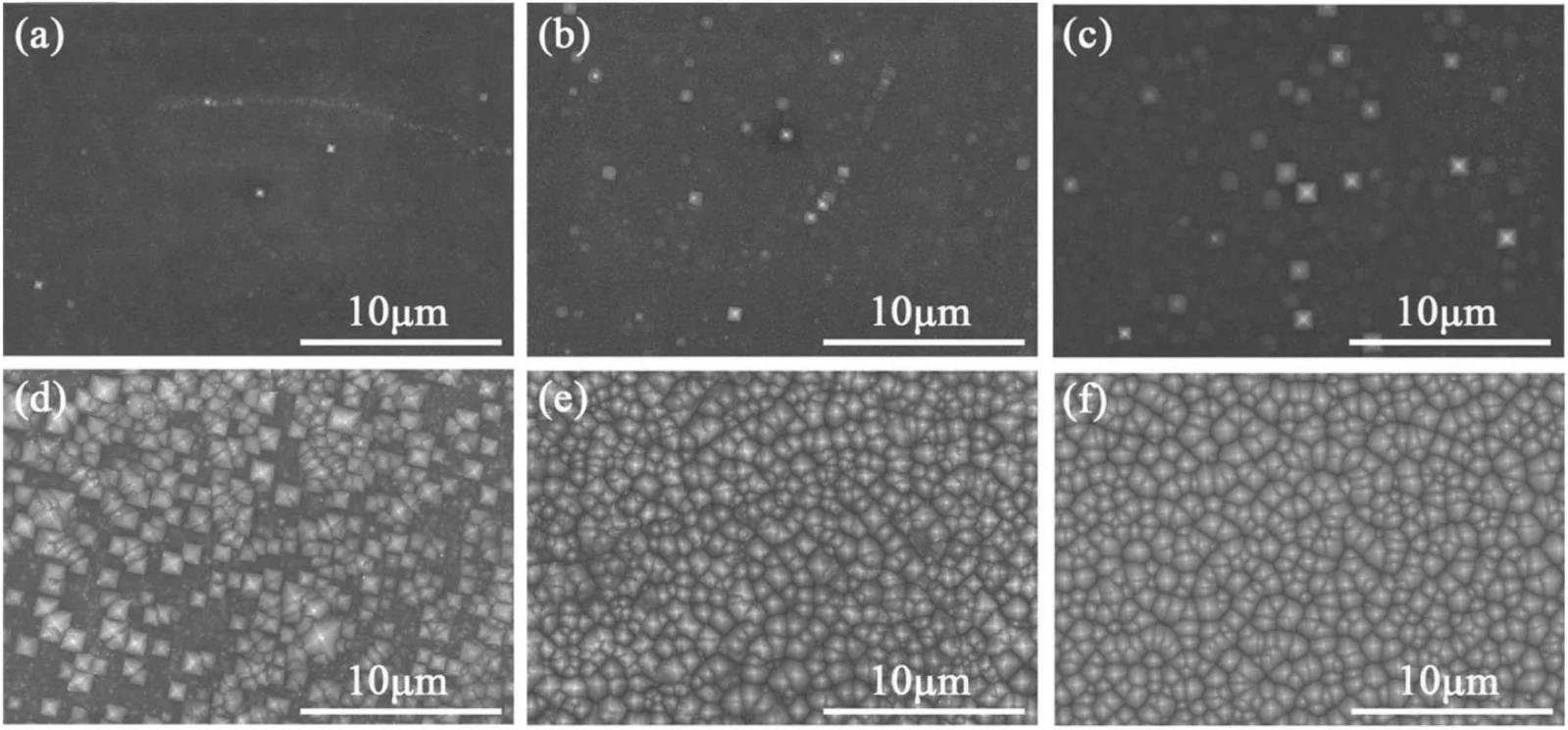
Surface morphology evolution on (100) p-Si wafers in aqueous I_2_–C_2_H_5_OH–HF (0.05 mol L^−1^/0.1 mol L^−1^/1.5 mol L^−1^) solution at 300 K. (a) 5 min. (b) 10 min. (c) 15 min. (d) 20 min. (e) 25 min. (f) 30 min. Reproduced with permission from ref. 23. Copyright 2019, The Electrochemical Society.

### 1.5. Polyhalide ions X_*n*_^−^ (X = Cl, Br, I)

Polyhalide ions have been known for a long time, especially those of iodine.^27^ Polychlorides and bromides were investigated much later.^28^ In contrast to molecular halogens, trihalide ions are chemically similar to hydroxide ions, because they are nucleophilic. The electrostatic potential of trihalides is anisotropic and therefore distributed unevenly (see Fig. 9).^29^ Like halogens, trihalide ions are σ-hole compounds (see Fig. 9, blue region), the negative charge is localized cylindrically around the main axis (red regions).^30^ The size of the sigma hole increases within the halogens, F ≪ Cl < Br < I, which consequently leads to greater stability of polyiodide anions compared to polyfluoride anions.^31^ The anisotropic charge distribution is stronger for the heavier halogens, which also leads to a higher nucleophilicity. Due to the uneven charge distribution, trihalides can interact with both nucleophiles (Nu^−^) and electrophiles (E^+^).^31^ With a nucleophile, the trihalide interacts *via* its σ-hole with a bond angle of 180°, and with an electrophile *via* the ring-shaped accumulations of negative partial charge with a bond angle of 90°.^29^

**Fig. 9:**
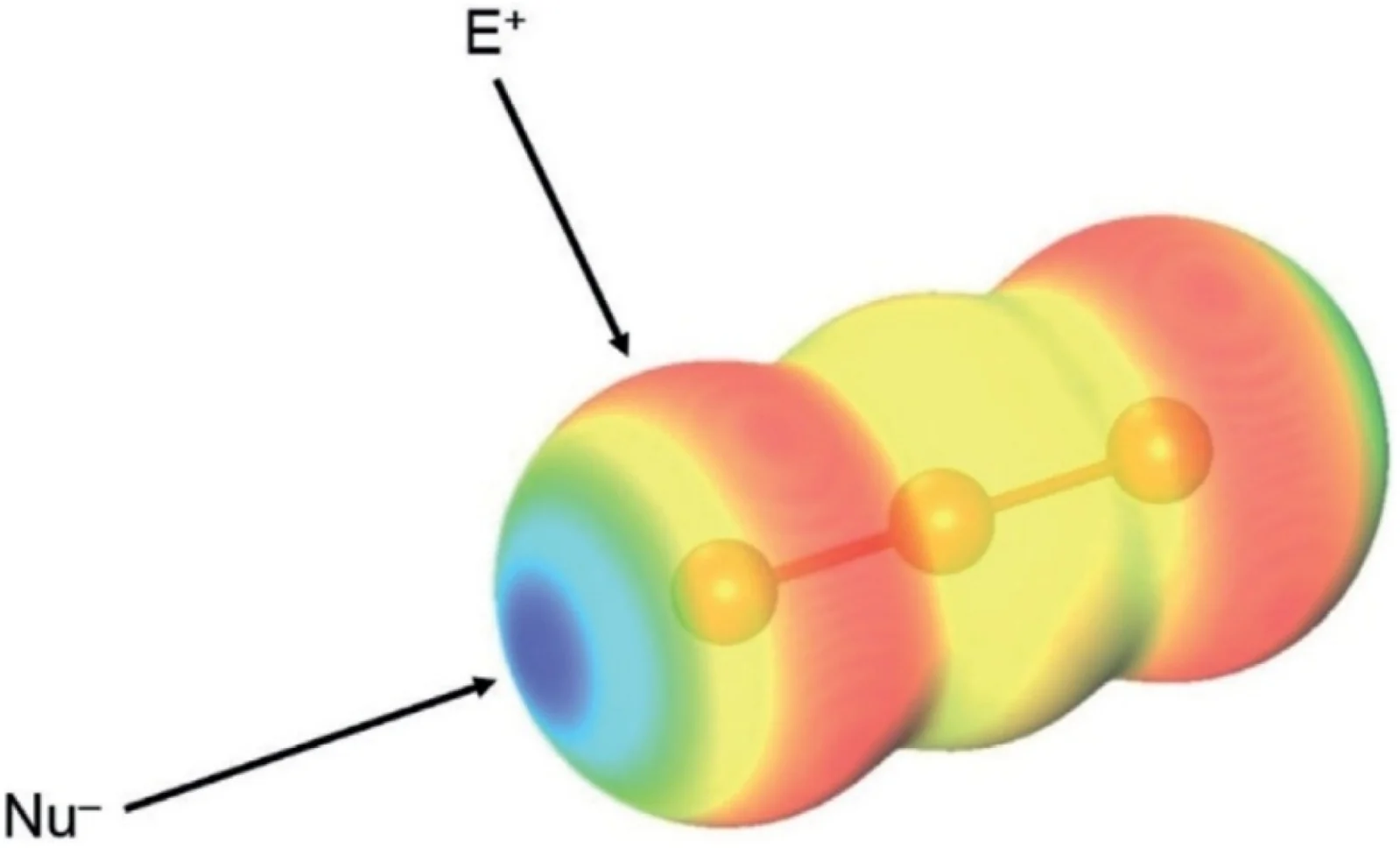
Electrostatic potential of the tribromide ion in the range from −0.175 a.u. (red) to −0.15 a.u. (blue), calculated on an isosurface of the electron density (0.0035 a.u.); B3LYP-D3/def2-TZVPP level. Reproduced with permission from ref. 31. Copyright 2019, John Wiley and Sons.

The tendency of trihalide ion formation increases from fluorine to iodine.^5^ In general, the trihalide formation is a reaction between a halogen (X_2_) and the corresponding halide (X^−^, see eqn (1)).

1 X_2_ + X^−^ ⇌ X_3_^−^

The equilibrium constant in aqueous solution for reaction ((1)) is 0.01 for chlorine, 18 for bromine, and 725 for iodine.^5^ As a result, the trichloride ion is only formed to a greater extent when a concentrated chloride-containing solution is saturated with Cl_2_.^32^ Tribromide and triiodide ions form even in dilute solutions.^5^ Alcoholic polyhalide solutions are more stable than aqueous solutions.^5^ Higher polyhalides are also known; for example, in the case of Br_2_, the pentabromide ion is formed if Br_2_ is added to tribromides (see eqn (2)). The equilibrium constant for reaction (eqn (2)) is 40 and the ion is V-shaped.^33^

2 Br_3_^−^ + Br_2_ ⇌ Br_5_^−^

The halogen–halogen bonds in tri- and polyhalides can be described as charge-shift bonds, because they are characterized by high electron delocalization and fluctuation indices, respectively.^34^ Polybromide ions exhibit high electrochemical conductivity, which can be attributed to a Grotthuss-like mechanism of charge transfer.^35^ According to the valence bond theory, trihalides can be described by mainly four resonant structures, showcasing the high electron delocalization (see eqn (3)).^34^

3 X_3_^−^ = X^−^X–X ⇌ X–XX^−^ ⇌ X^−^X^+^X^−^ ⇌ X˙X^−^X˙

## 2. Results and discussion

### 2.1. Formation of X_*n*_^−^ in HX solutions

Aqueous solutions of the polyhalides can be investigated *via* Raman spectroscopy, showing characteristic polyhalide bands.^36^ Oxidation experiments were carried out with H-terminated Si wafer fragments and solutions of the halogens in the corresponding hydrohalic acid, respectively. Fig. 10 shows Raman spectra of the corresponding solutions, together with a spectrum of chlorine water (top, light green line). All band positions, compared to literature data, can be found in Table 1. Photos of the solutions can be found in the SI (see Fig. S1). The full spectra can also be found in the SI (see Fig. S2).

**Fig. 10:**
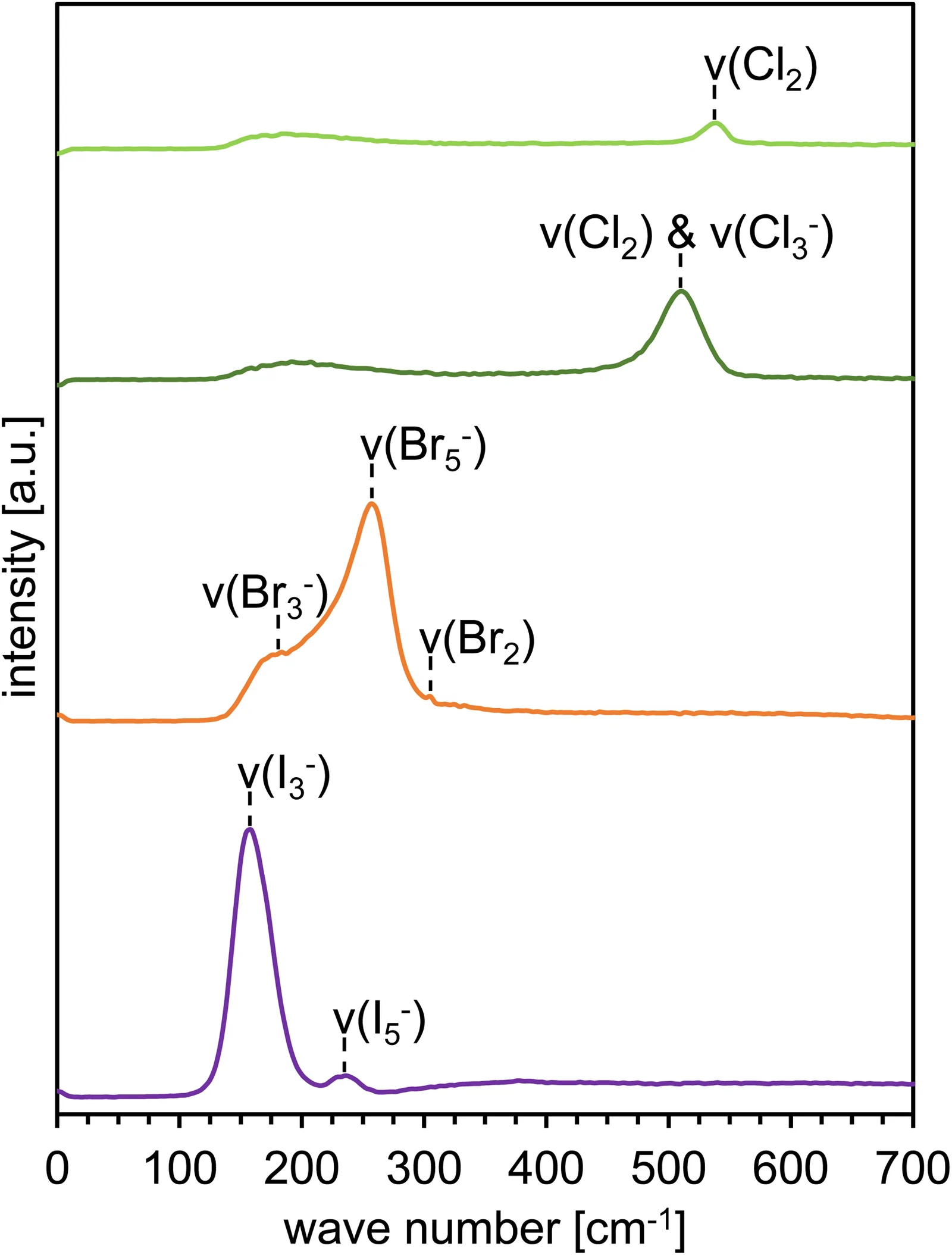
Raman spectra of a saturated solution of Cl_2_ in H_2_O (light green), a saturated solution of Cl_2_ in 37% HCl (dark green), a solution of Br_2_ in 48% HBr (orange) and a solution of I_2_ in 57% HI (purple).

**Table 1:** Observed halogen RAMAN signals compared to the literature

Species	Observed wave number (cm^−1^)	Literature wave number (cm^−1^)	Literature
Cl_2_	540	530	36
Cl_3_^−^	509	268	36
Br_2_	305	301	36
Br_3_^−^	175	164	36
Br_5_^−^	257	253	29
I_2_	—	207	36
I_3_^−^	159	149	36
I_5_^−^	228	210	37

When Cl_2_ is introduced into a concentrated HCl solution, a Raman band appears at lower wave numbers compared to chlorine water. Due to trichloride ion formation, a second band appears, merging with the band for dissolved chlorine.^32,35^ The observed band position differs significantly from the literature values for trichloride, which is due to the different chemical environment. The literature values were determined on solid trichloride salts with organic counter ions.^35,36^ In this experiment, the aqueous environment influences the band position, shifting it only slightly towards lower wave numbers. It is assumed that chlorine is mainly physically dissolved and only partially present as trichloride ions.^32^ Therefore, two bands should be visible. The applied Raman spectrometer has a resolution too low to resolve the bands of chlorine and trichloride.

The Raman spectrum of Br_2_ in HBr shows bands of tribromide and pentabromide ions.^29,36^ At 305 cm^−1^ a very small band of molecular bromine can be seen.^36^ The Raman spectrum of a solution of I_2_ in concentrated HI shows bands of tri- and pentaiodide ions, while a band for molecular iodine at 207 cm^−1^ is not observed.^36,37^

### 2.2. Oxidation of Si–H-surfaces with X_2_ and X_3_^−^-containing solutions

The Si–H vibration is IR-active, an analysis of treated wafers *via* diffuse reflectance infrared Fourier transform spectroscopy (DRIFTS) is feasible to identify surface species.^17^ The above-mentioned solutions of the halogens in their corresponding hydrohalic acid were used for the oxidation of H-terminated Si wafer surfaces. DRIFT spectra of H-terminated Si wafer fragments before and after oxidation are shown in Fig. 11. The full DRIFT spectra can be found in the SI (see Fig. S3–S9).

**Fig. 11:**
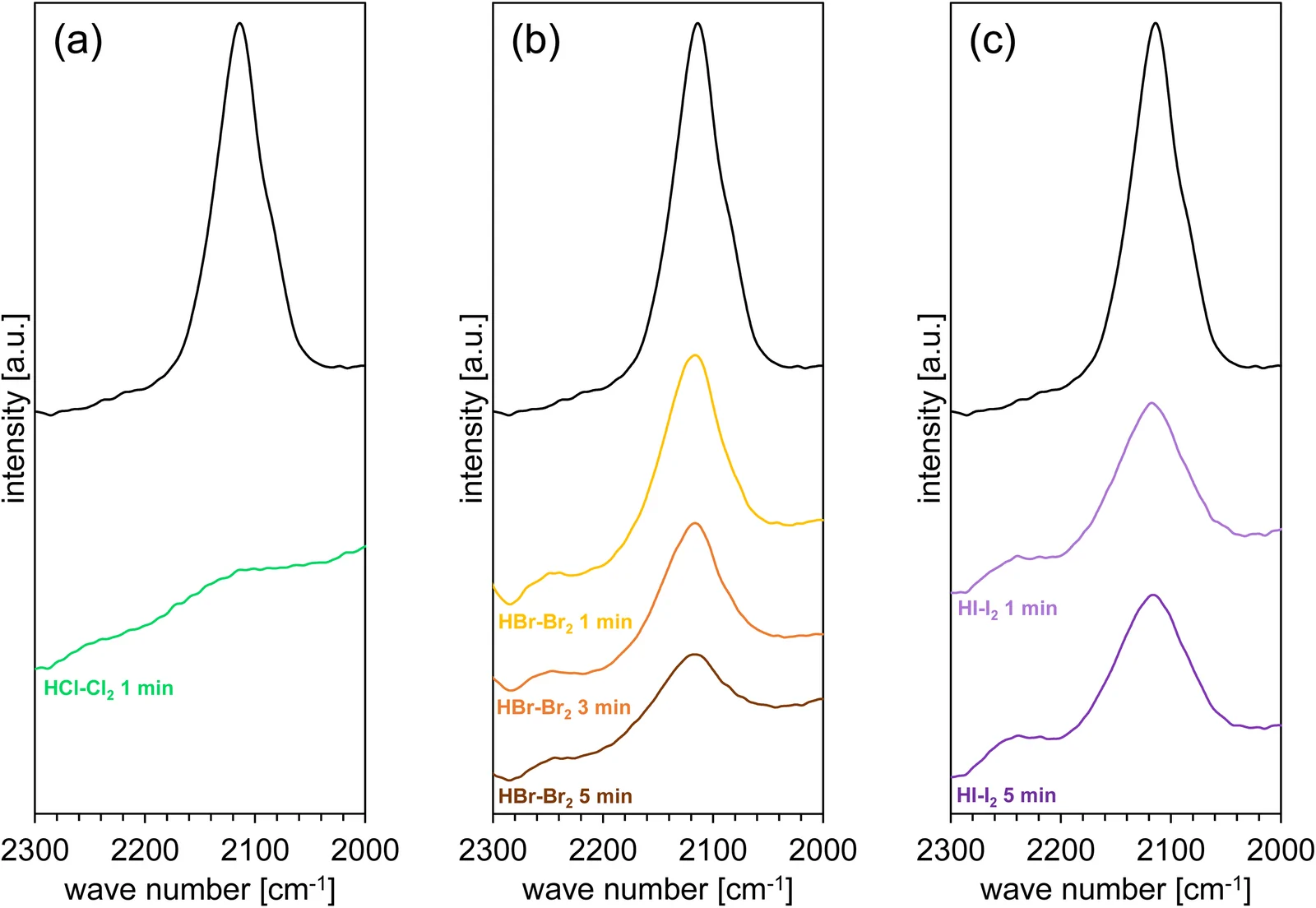
(a) DRIFT spectra of a H-terminated Si surface (black) and after 1 minute of treatment in a HCl–Cl_2_ solution (green). (b) DRIFT spectra of a H-terminated Si surface (black), after treatment in a HBr–Br_2_ solution (yellow: 1 min, orange: 3 min, brown: 5 min). (c) DRIFT spectra of a H-terminated Si surface (black), after treatment in a HI–I_2_ solution (light purple: 1 min, dark purple: 5 min).

In previous oxidation experiments with aqueous solutions of Cl_2_ and Br_2_, we observed that the intensity of the Si–H vibration is reduced significantly, but it does not disappear completely.^17,18^ A very weak Si–H band remains, while weak bands for Si(O)_*x*_–H species are also observed.^17^ The oxidation experiment with Cl_2_ in concentrated HCl (see Fig. 11a) also shows this behavior. We assume that dissolved chlorine is responsible for the abstraction of hydrogen and the disappearance of the Si–H band. The formation of Si–X functions removes electron density from the terminal Si atom. Thus, the protic character of the remaining hydride H atoms is increased as more Si–H functions are replaced by Si–X functions. We assume that if enough Si–H functions were replaced by Si–X functions, the remaining H atoms could take on such a protic character that they become completely unreactive towards the electron-deficient halogen. This might explain why the Si–H band is never removed completely.

The oxidation experiments with solutions of Br_2_ in 48% HBr and I_2_ in 57% HI are more interesting. With Br_2_, a distinct Si–H band with reduced intensity remains after 1 minute of reaction time (see Fig. 11b). We repeated the oxidation experiment with treatment times of up to 5 minutes and observed a gradual decrease in the intensity of the Si–H band. At the same time, bands for Si(O)_*x*_–H species are observed around 2250 cm^−1^.^36^ This indicates the oxidation of near-surface Si–Si bonds, possibly by water, in addition to the oxidation of terminal Si–H bonds. After 5 minutes of treatment, a distinct Si–H band is still visible. Compared to HCl–Cl_2_ solutions, HBr–Br_2_ solutions exhibit reduced reactivity toward Si–H bonds. With long reaction times, the intensity of the Si–H vibration decreases, which may be due to a small amount of molecular Br_2_ in the oxidation solution. If the same experiment is performed with a saturated solution of I_2_ in concentrated HI, a similar decrease in intensity is observed, together with the formation of some Si(O)_*x*_–H bands. However, the intensity of the Si–H band does not decrease further when the treatment time is increased. The overall intensity decrease is similar to a treatment in concentrated HNO_3_.^18^ Since HNO_3_ does not attack the H-termination and only oxidizes rearward Si–Si bonds, we assume that the Si–H termination is inert towards polyhalide solutions.^38^ It is also known from the literature that the Si–H termination is stabilized at low pH values.^39^ The fact that HBr and HI are very strong acids could contribute to the observed effects. Nevertheless, it appears that only the HCl–Cl_2_ solution attacks the Si–H bonds at a high rate. This solution contains a significant proportion of dissolved Cl_2_, whereas the other solutions mainly contain tri- and polyhalides.

### 2.3. Presumed reaction pathways

On H-terminated silicon surfaces, terminal silicon atoms are partly positively charged, while hydride hydrogen saturates the dangling bonds.^5^ The electron-rich hydrogen atoms can be attacked by electron-deficient halogens, forming the corresponding hydrohalic acid and another halide ion (see Fig. 12). This reaction pathway does not involve any energetically unfavorable transition states and can be described as an accelerated S_N_1 reaction known from organic chemistry. We assume that isotropic etching in HF–X_2_ (X = Cl, Br, I) solutions proceeds *via* this path. The silicon atoms are temporarily terminated with halogen atoms (*e.g.* bromine), polarizing the rearward Si–Si bonds. We observed Si–Cl and Si–Br species in previous XPS measurements, where we analyzed oxidized and etched Si surfaces.^6,18,19^ After oxidation with HF-free trichloride or -bromide solutions, a mixed surface termination consisting of Si–H- and Si–X-groups is observed in addition to a very high oxygen content. After etching, Si surfaces are mainly H-terminated, but the hydrogen termination is almost completely removed by the halogens Cl_2_ and Br_2_. Due to their instability towards hydrolysis, the Si–X bonds must be broken quickly. In HF solutions, an exchange to Si–F groups appears also likely, as silicon is very fluorophilic.

**Fig. 12:**
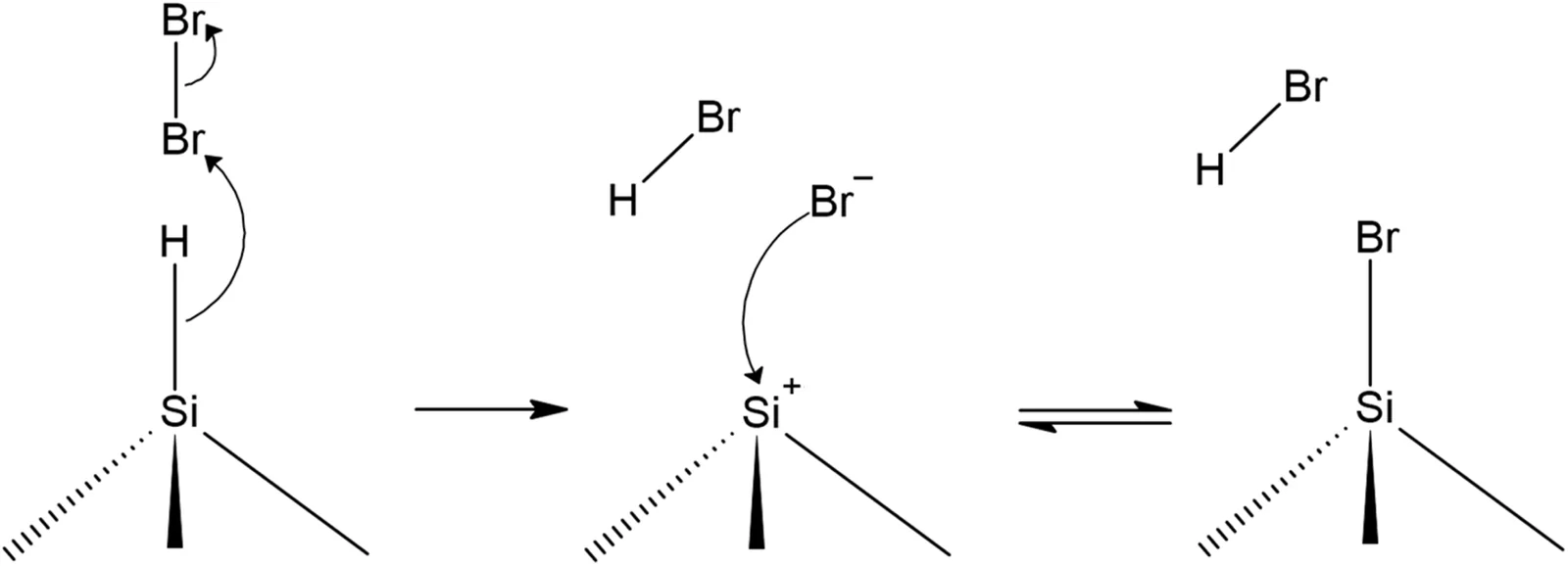
S_N_1-like reaction scheme for the attack of Br_2_ on a H-terminated (111)Si surface. Analogous reactions may occur on (100)- as well as other Si surface orientations.

If polyhalides are the main oxidizer, the surface oxidation proceeds *via* a different path. An attack of polyhalides on hydride hydrogen seems unlikely because of their negative charge. An attack on the positively charged silicon atoms is more feasible, because polyhalides exhibit nucleophilic properties. This is comparable to the attack by hydroxide ions known from alkaline etching (see Fig. 4). This reaction pathway can be understood as an accelerated S_N_2 reaction, since the electron-rich leaving group (hydride hydrogen) is actively removed by the electrophilic Br_2_. In contrast to the classic S_N_2 reaction, no true backside attack can occur due to the Si bulk; the attack of the polyhalide and the secession of the leaving group must occur on the same side. A pentavalent silicon environment is formed as a transition state (see Fig. 13, step II), leading to the observed anisotropic etching behavior. The cleavage of a Br–Br bond liberates a Br_2_ molecule, which can directly abstract the electron-rich terminal H atom. This temporarily terminates the Si surface with Br atoms.^18^

**Fig. 13:**
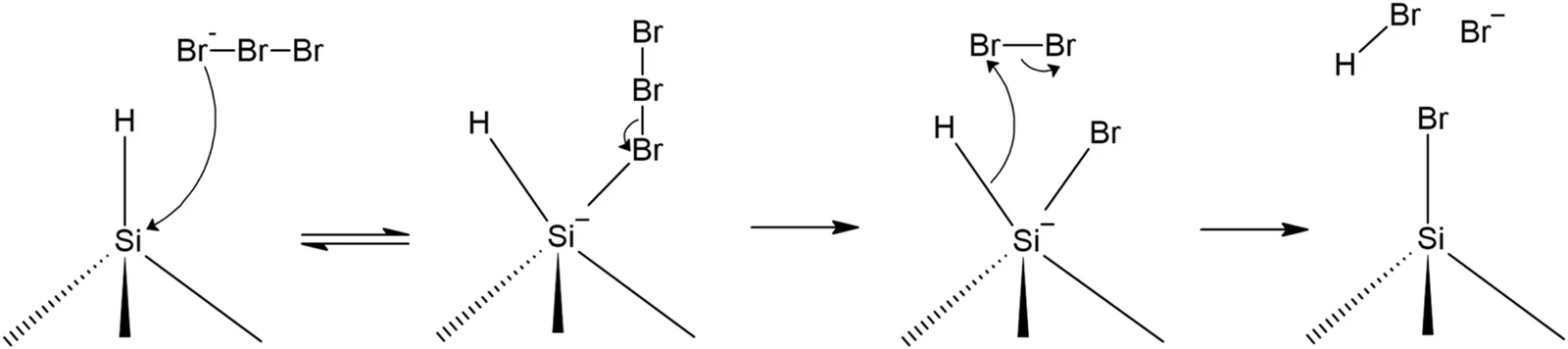
S_N_2-like reaction scheme for the attack of Br_3_^−^ on a H-terminated (111)Si surface. Analogous reactions may occur on (100)- as well as other Si surface orientations.

Fig. 14 shows a comparison of the different oxidation mechanisms that lead to anisotropic silicon dissolution. Although the further course of the reaction after oxidation differs significantly (abstraction of the oxidized Si atom by H_2_O or HF), the oxidation pathways are very similar.

**Fig. 14:**
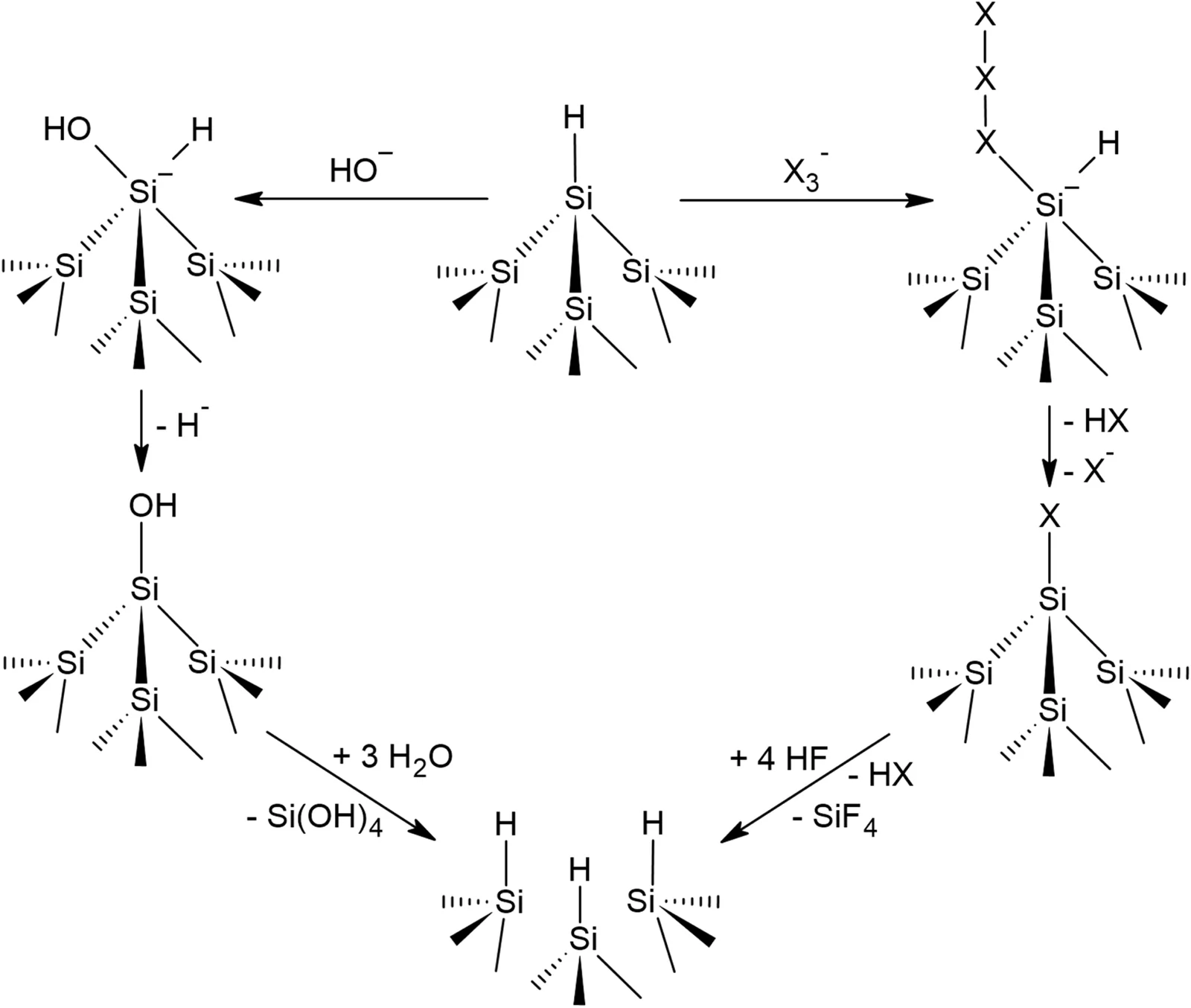
Comparative reaction scheme showing the difference between the oxidation step in alkaline etching and the oxidation step in acid etching with polyhalides.

## 3. Conclusions

This study investigated the oxidation behavior of polyhalide solutions toward H-terminated Si wafer surfaces, drawing parallels to reactions in alkaline solutions.

Aqueous polyhalide solutions do not remove the hydrogen termination compared to solutions of molecular halogens. This is due to the conversion of electrophilic X_2_ molecules to nucleophilic anionic species, which significantly alters the interaction with the silicon surface. This enables anisotropic silicon dissolution in HF solutions, akin to the behavior of hydroxide ions in alkaline etchants.

Polyhalide formation explains the tendency of Br_2_- and I_2_-containing HF solutions to quickly change the etching regime towards anisotropic silicon dissolution although initially polishing the surface. The polishing regime in Cl_2_-containing HF solutions is much more resilient, texturing only occurs if HCl is added deliberately.^4,26^ This correlates with the formation tendency of trichloride ions being significantly less favored compared to the trihalides of the heavier halogens.^5,40^

Thus, the toolbox for wet-chemical texturing of monocrystalline silicon surfaces was expanded by the introduction of acidic polyhalide-based HF solutions. The findings reveal that polyhalide ions act as functional analogues of hydroxide ions in acidic etching systems, facilitating anisotropic etching through a nucleophilic attack on Si surface atoms and forming pentacoordinated transition states. However, the complexity of polyhalide formation, stability, and reactivity introduces challenges in achieving homogeneous texturing on full wafer scales.

This study is a foundational mechanistic investigation and therefore theoretical calculations should be applied to validate the proposed surface reactions and compare the trihalide reaction behavior with other species, especially halogens X_2_ and hydroxyl ions. There are, however, atomistic Monte-Carlo simulations for the dissolution of Si surfaces done by Gosalvez *et al.* in 2001 for anisotropic etching behavior in alkaline etching media.^41^ These support the idea of the nucleophilic S_N_2 like substitution proposed by Baum *et al.*^8^

Besides, detailed quantitative concentration-dependent studies should be performed in the future in order to correlate trihalide content with Si surface texture anisotropy. However, determination of trihalide concentrations is not trivial for all three considered halogens requiring the selection of suitable analytical methods and careful calibration measures.

## 4. Experimental

### 4.1. Chemicals and materials

For the oxidations experiments, we used (100) Si wafer pieces of approximately 10 × 10 mm ((100) orientation, boron doped, as-cut diamond wire sawn, thickness of 180 µm, resistivity of 1.75 Ω cm^−1^, SolarWorld Industries Thüringen GmbH, Germany). The wafer pieces were pretreated by etching in HF–HNO_3_ solution (HF: 50 wt%, VLSI, Honeywell; HNO_3_: 69 wt%, VLSI, Honeywell). For the oxidation experiments, we used HCl (37 wt%, analytical reagent grade, Fisher Scientific), HBr (48 wt%, analytical reagent grade, Thermo Fisher Scientific), HI (57 wt%, p.a., Merck, old stock with visible iodine formation), Cl_2_ (2.8, Air Liquide) & Br_2_ (99.6%, p.a., Thermo Fisher Scientific).

### 4.2. Oxidation procedure

**Caution!** Etching experiments with hydrofluoric acid must be performed in a HF-approved fume hood with HF-resistant laboratory equipment.

To H-terminate the silicon surface, as-cut silicon wafer pieces of approx. 10 × 10 mm were pretreated by etching in an aqueous mixture containing 11.7 mol L^−1^ HF and 3.9 mol L^−1^ HNO_3_ for 5 min. After etching, the wafer pieces were rinsed with DI water and dried with pressurized air. The obtained H-terminated wafer pieces were then placed into the oxidation solution, rinsed with DI water and dried with pressurized air. The experiments were conducted in 50 mL perfluoroalkoxy copolymer (PFA) beakers with 300 rpm of stirring (PTFE coated stir bar).

### 4.3. Characterization of the oxidation solutions

The solutions were investigated by Raman spectroscopy. An Ocean Insight QEP05509 Raman spectrometer with a 785 nm laser source (499 mW) was used. The spectra were acquired at an integration time of 10 s and averaged from 3 scans. The focal point of the laser was set to 7 mm in front of the measuring probe window. The measurement was conducted directly through the wall of the PFA sample container.

### 4.4. Characterization of treated silicon wafers

A Thermo Fisher Scientific Nicolet iS50 FTIR spectrometer with Smart Collector Avatar accessory was used for the DRIFTS measurements of treated silicon wafers. For each DRIFTS-measurement, 64 scans with a resolution of 0.96 cm^−1^ were collected and averaged.

## Author contributions

N. S.: data curation, formal analysis, investigation, methodology, validation, visualization, writing – original draft. N. Z.: data curation, formal analysis, supervision, validation, writing – review and editing. A. S.: conceptualization, funding acquisition, methodology, project administration, resources, supervision, writing – review and editing. E. K.: conceptualization, funding acquisition, project administration, resources, supervision, writing – review and editing.

## Conflicts of interest

There are no conflicts to declare.

## Supplementary Material

RA-016-D6RA05037A-s001

## Data Availability

The data supporting this article have been included as part of the supplementary information (SI). Supplementary information is available. See DOI: https://doi.org/10.1039/d6ra05037a.
