# Supplementary material for: Mechanistic insights into the acidic wet-chemical silicon etching in HF–HX–X2 solutions, X = Cl, Br, I
Source: RSC Adv. 2026 Aug 21;16(44):54639–48. doi: 10.1039/d6ra05037a (PMC13494950; doi:10.1039/d6ra05037a)
Supplement: Supplementary file 1 [file RA-016-D6RA05037A-s001.pdf]

Supplementary Information (SI) for RSC Advances.
This journal is © The Royal Society of Chemistry 2026

Supplementary Information

Mechanistic insights into the acidic wet-chemical silicon etching in HF-HX-X_2 solutions, X = Cl, Br, I

Nils Schubert[a,b], Niklas Zomack[a,b], André Stapf[a,b], Edwin Kroke[a,b,]*

a) Institute of Inorganic Chemistry, Technische Universität Bergakademie Freiberg, Leipziger Str. 29, D-09599 Freiberg, Germany

b) Centre for Efficient High-Temperature Material Conversion, Technische Universität Bergakademie Freiberg, Winklerstr. 5, D-09599 Freiberg, Germany

*Corresponding author: Prof. Dr. Edwin Kroke, edwin.kroke@chemie.tu-freiberg.de

Contents

1 Photos of the oxidation solutions 2
2 RAMAN spectra 2
3 DRIFT spectra 3

1 Photos of the oxidation solutions

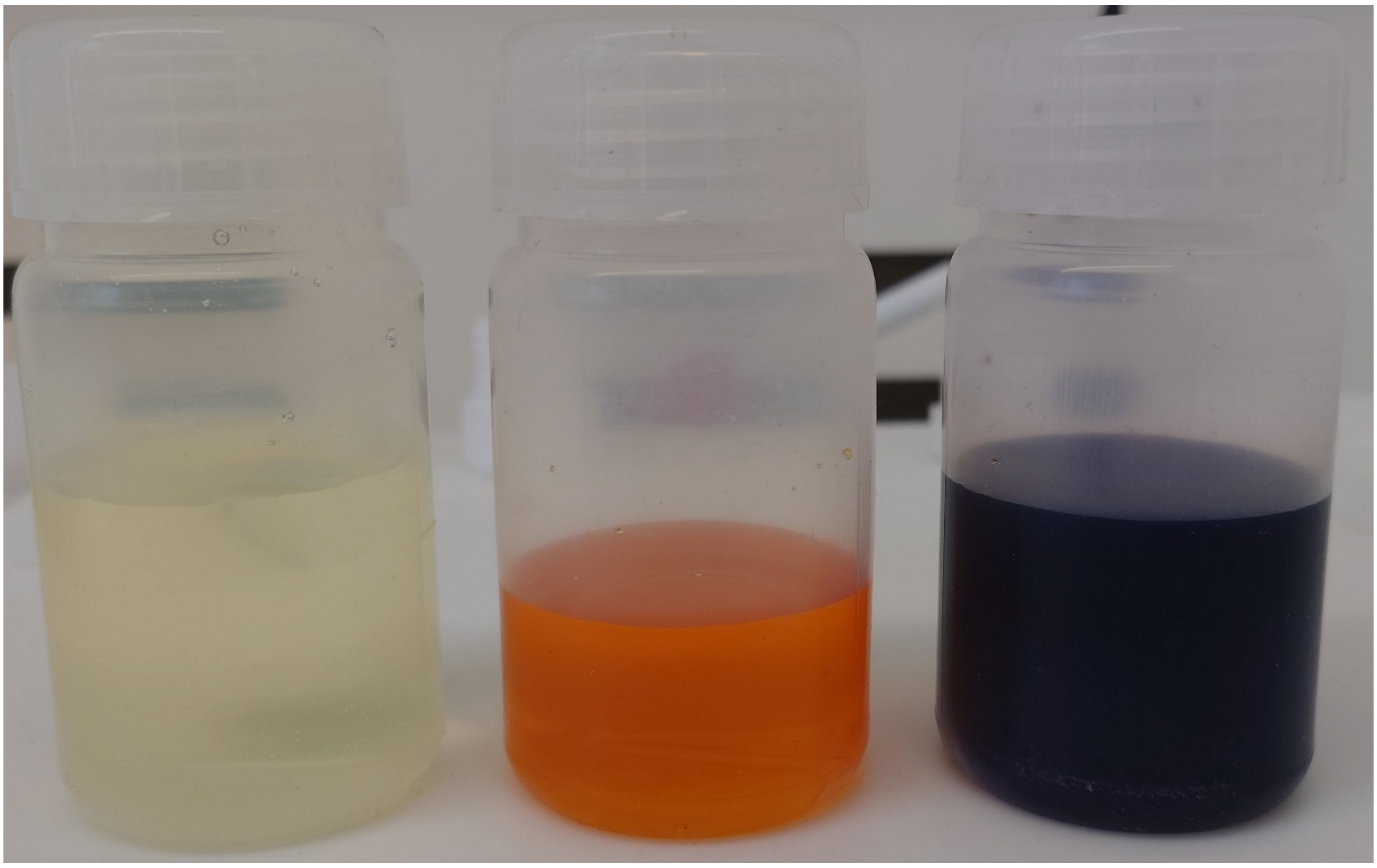

Figure S1: *Photos of the solutions used for the oxidation experiments, left: saturated solution of Cl_2 in 37% HCl, middle: solution of Br_2 in 48% HBr (62,5 mmol L^{-1}), right: saturated solution of I_2 in 57% HI.*

2 RAMAN spectra

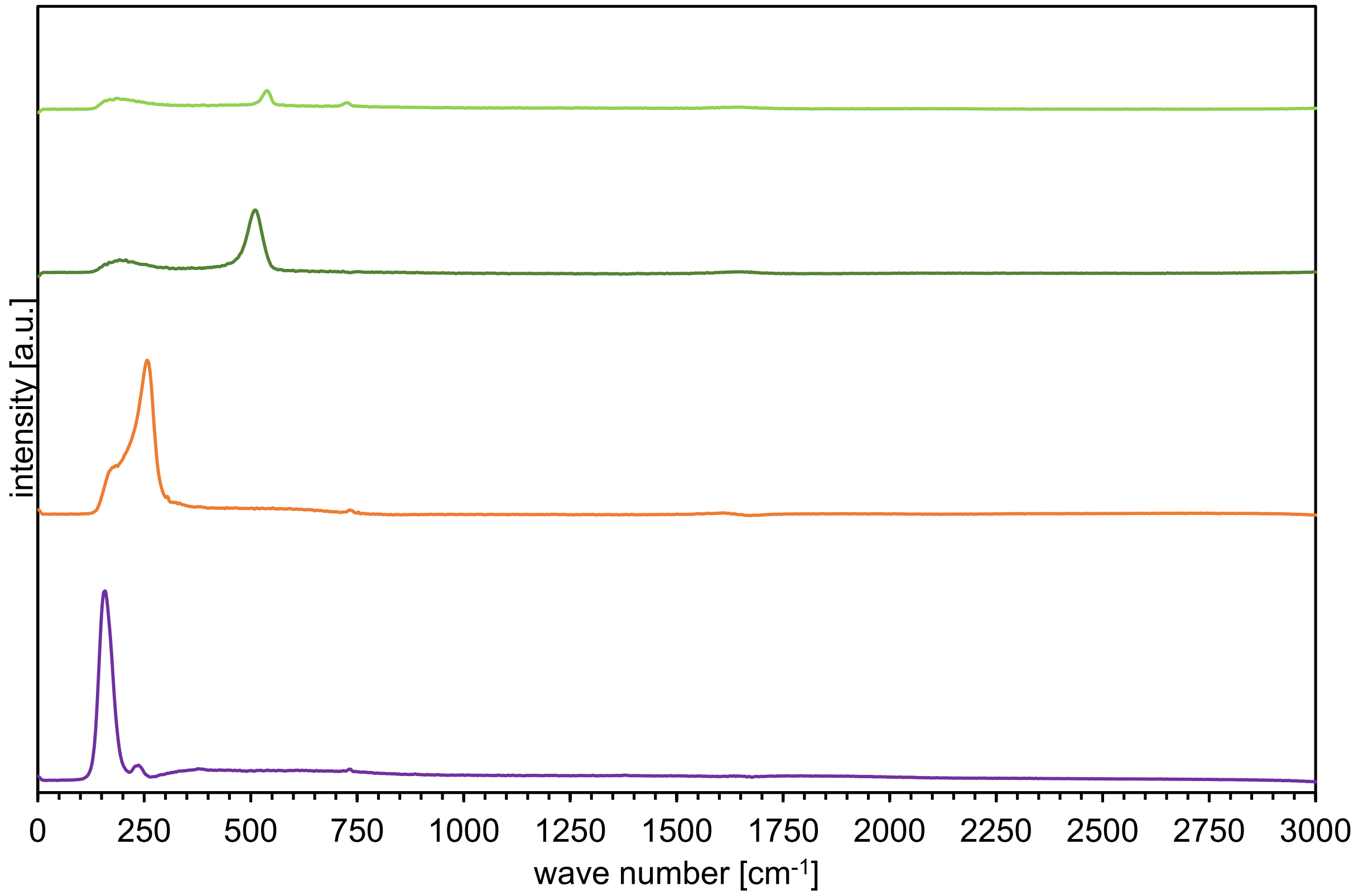

Figure S2: *RAMAN spectra of a saturated solution of Cl_2 in H_2O (light green), a saturated solution of Cl_2 in 37% HCl (dark green), a solution of Br_2 in 48% HBr (orange) and a solution of I_2 in 57% HI (purple).*

3 DRIFT spectra

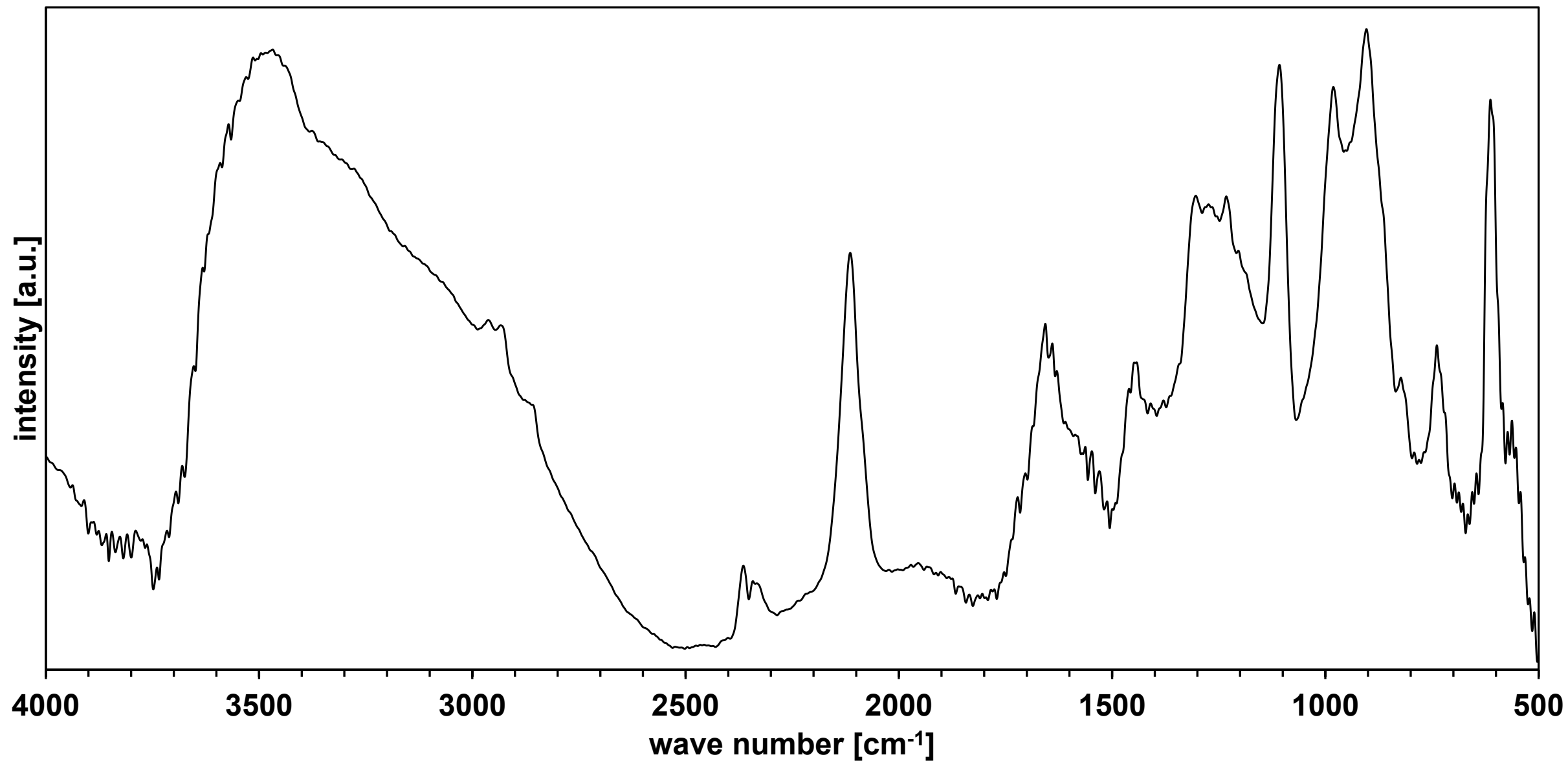

Figure S3: *DRIFT spectrum of a H-terminated Si surface.*

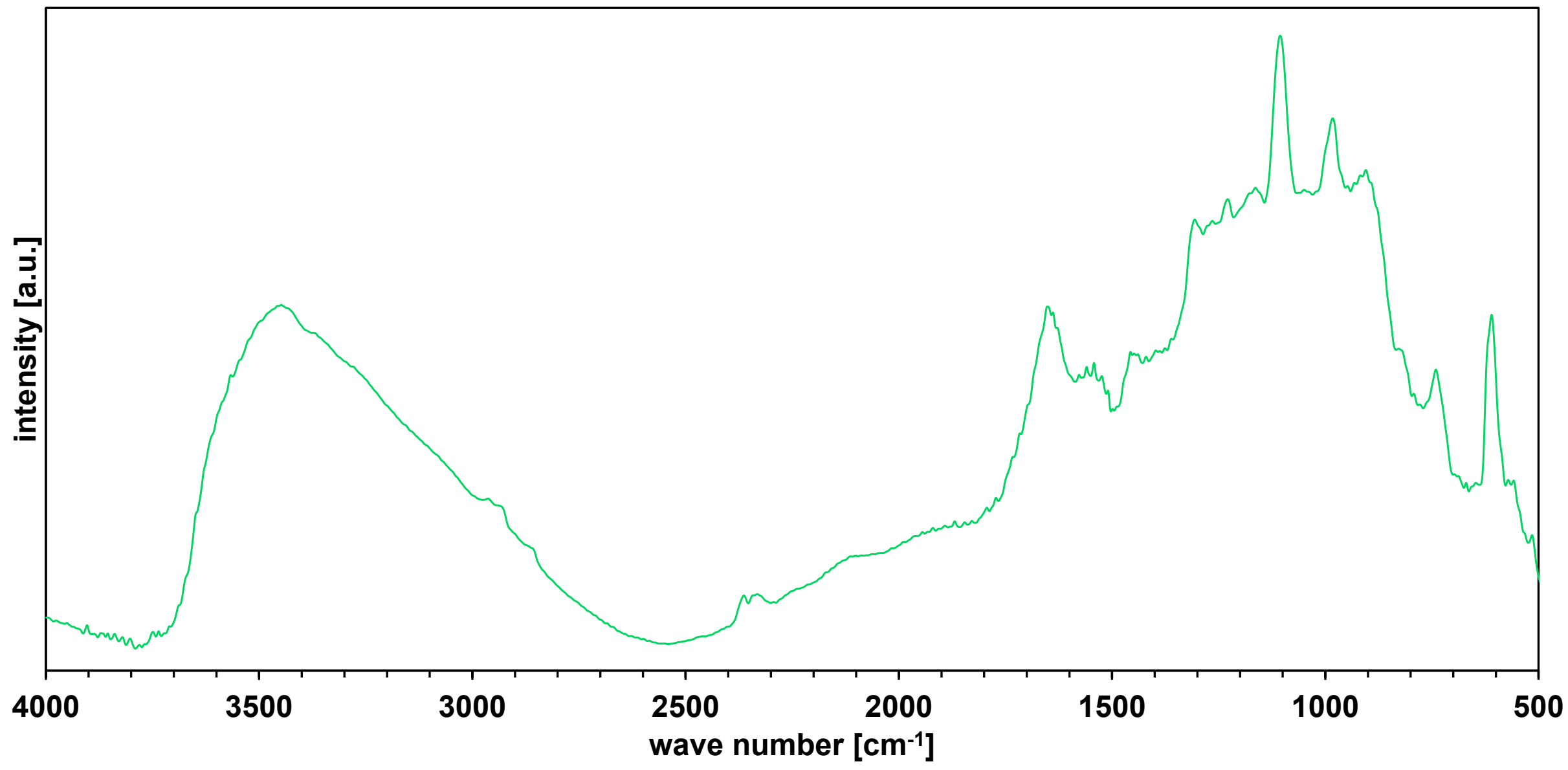

Figure S4: *DRIFT spectrum of a H-terminated Si surface after 1 minute of treatment in a HCl-Cl_2 solution.*

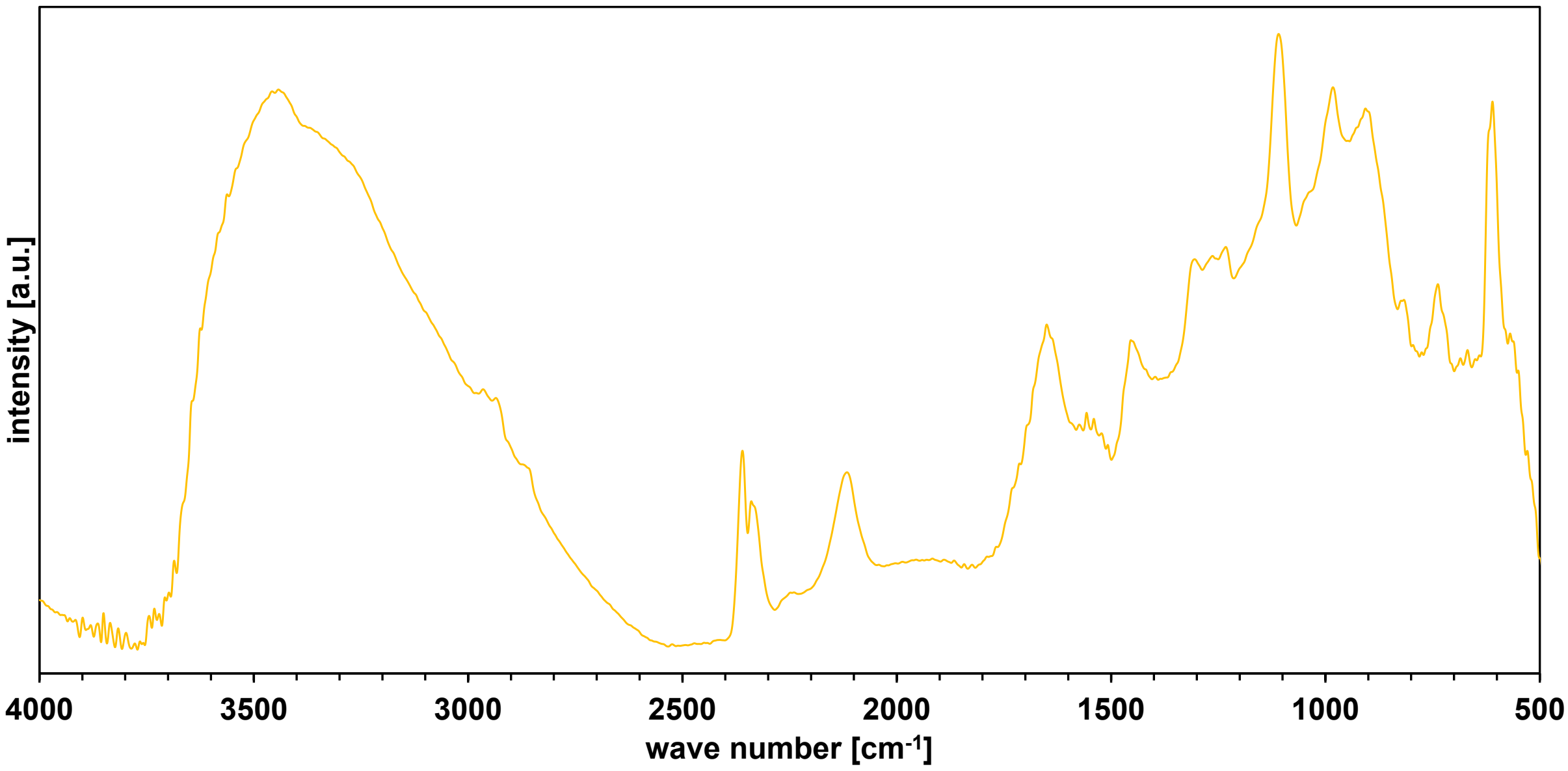

Figure S5: *DRIFT spectrum of a H-terminated Si surface after 1 minute of treatment in a HBr-Br_2 solution.*

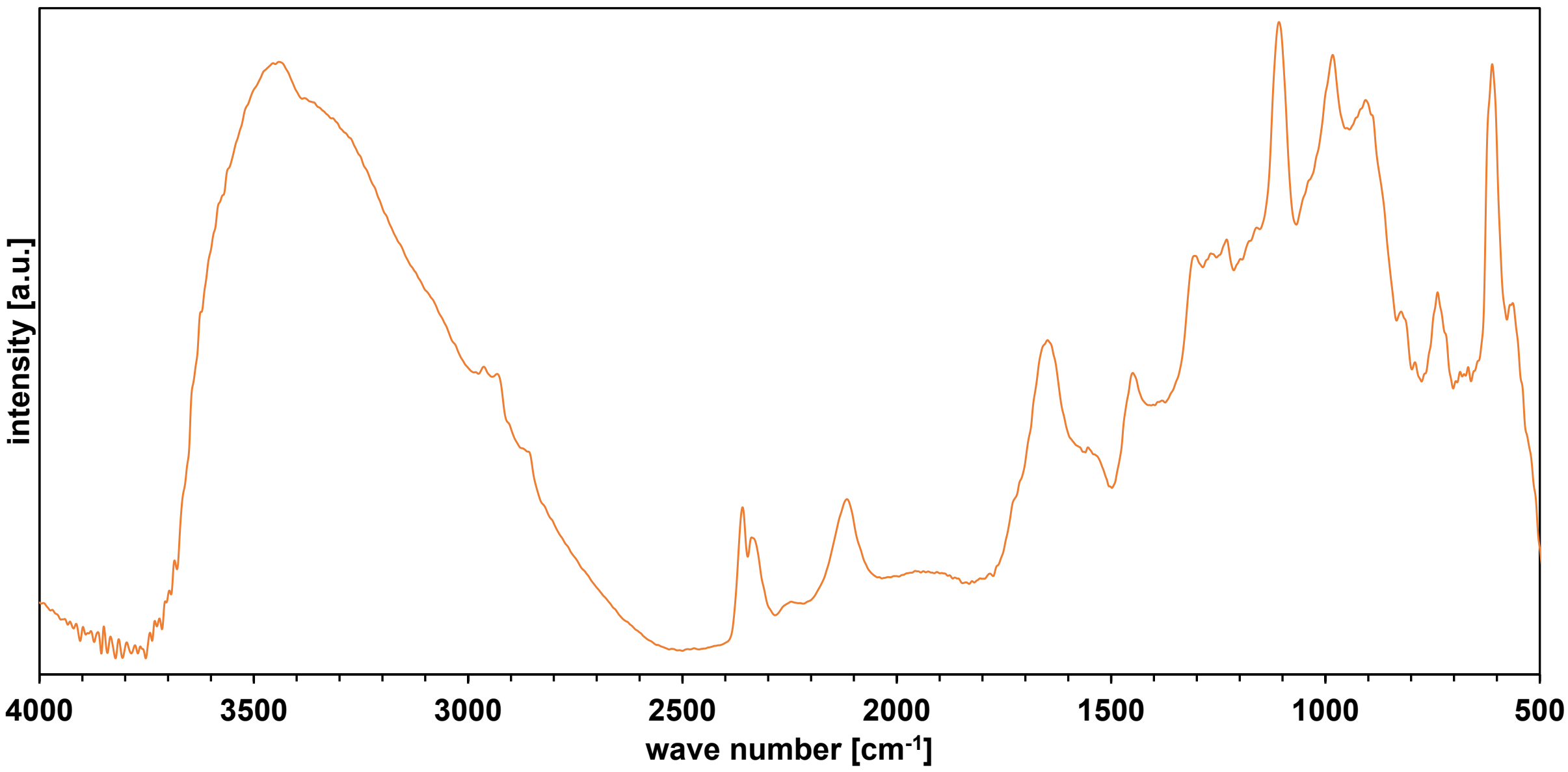

Figure S6: *DRIFT spectrum of a H-terminated Si surface after 3 minutes of treatment in a HBr-Br_2 solution.*

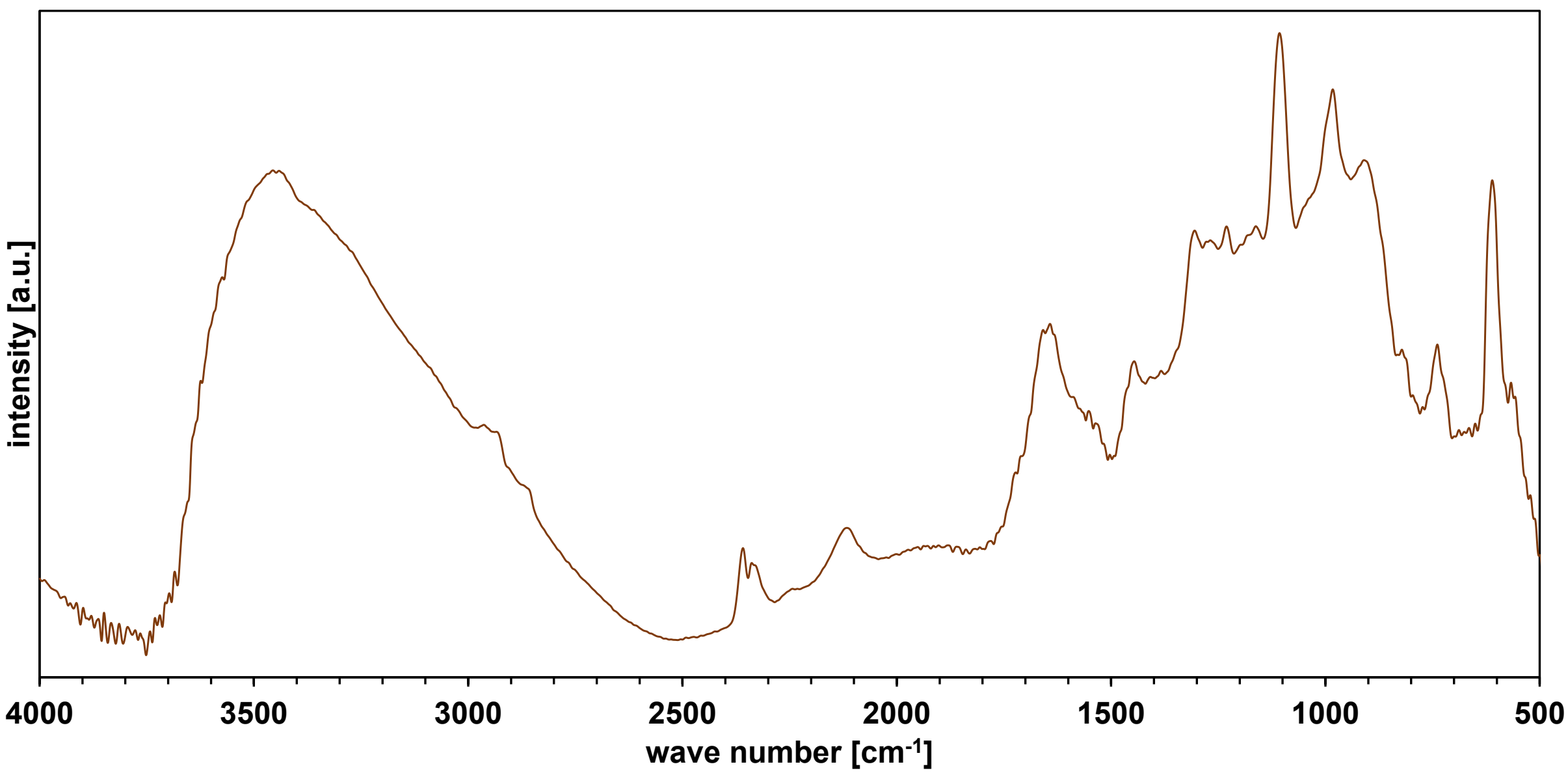

Figure S7: *DRIFT spectrum of a H-terminated Si surface after 5 minutes of treatment in a HBr-Br_2 solution.*

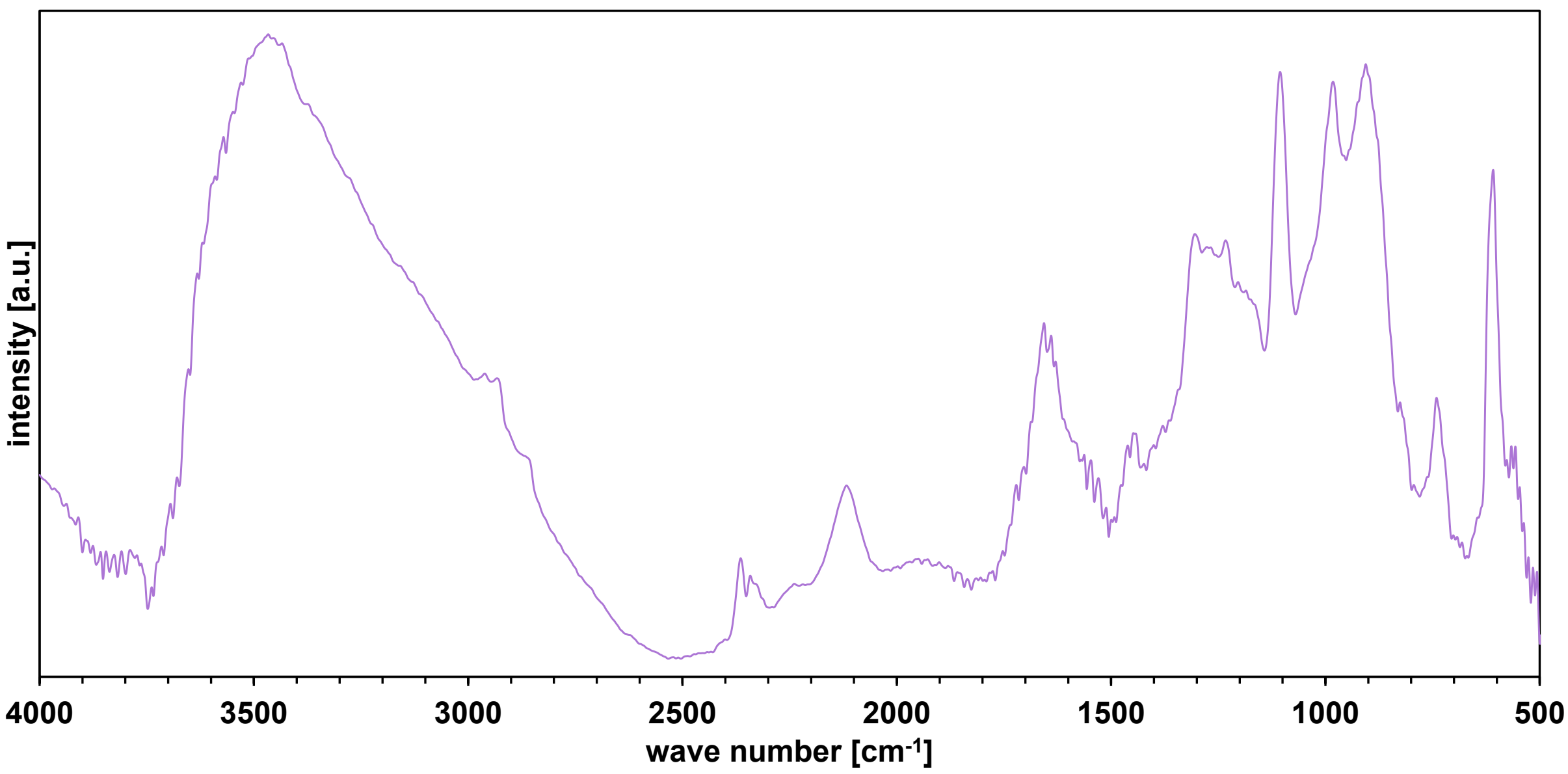

Figure S8: *DRIFT spectrum of a H-terminated Si surface after 1 minute of treatment in a HI-I_2 solution.*

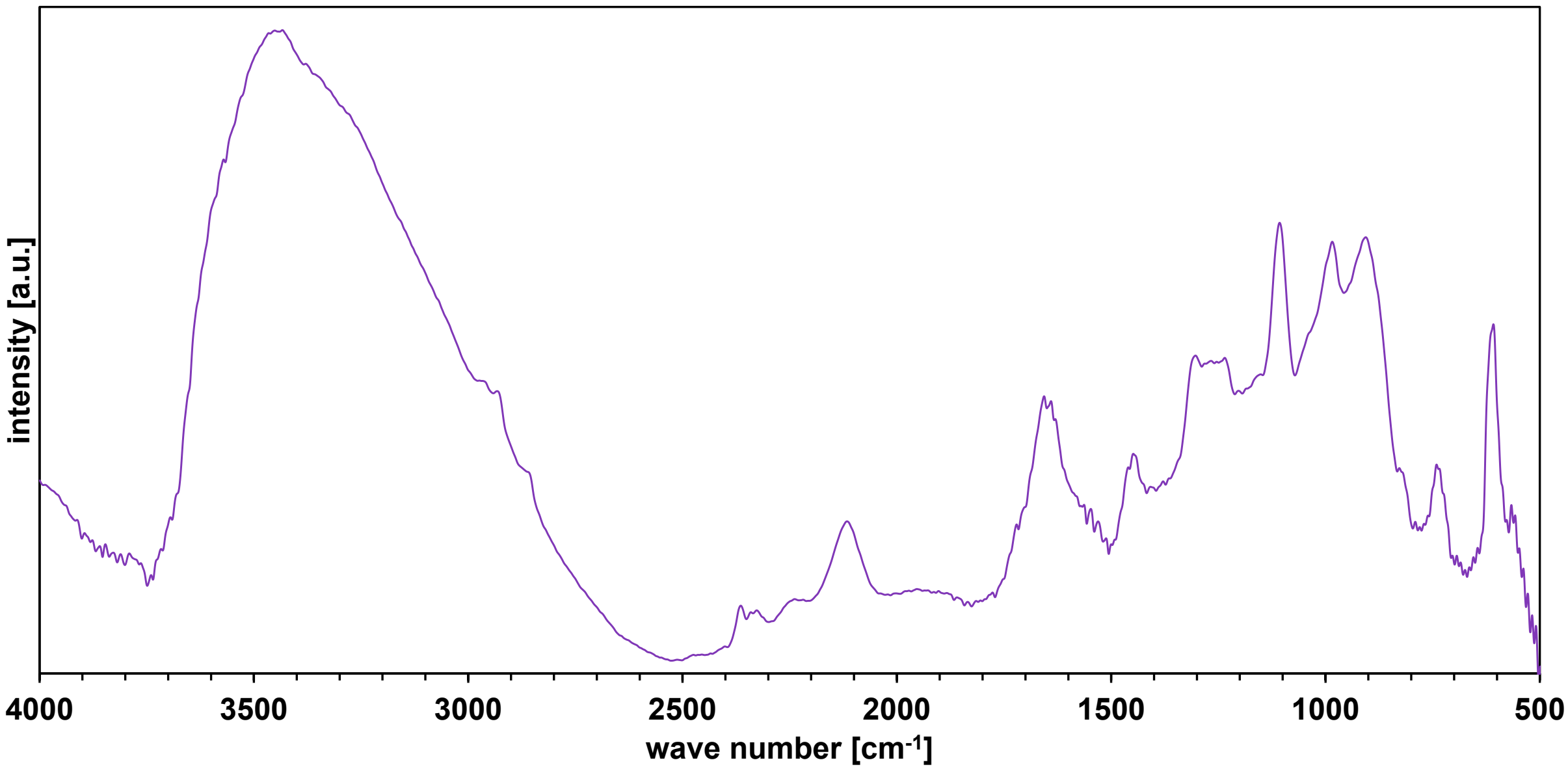

Figure S9: *DRIFT spectrum of a H-terminated Si surface after 5 minutes of treatment in a HI-I_2 solution.*
